# Five state factors control progressive stages of freshwater salinization syndrome

**DOI:** 10.1002/lol2.10248

**Published:** 2023-02-01

**Authors:** Sujay S. Kaushal, Paul M. Mayer, Gene E. Likens, Jenna E. Reimer, Carly M. Maas, Megan A. Rippy, Stanley B. Grant, Ian Hart, Ryan M. Utz, Ruth R. Shatkay, Barret M. Wessel, Christine E. Maietta, Michael L. Pace, Shuiwang Duan, Walter L. Boger, Alexis M. Yaculak, Joseph G. Galella, Kelsey L. Wood, Carol J. Morel, William Nguyen, Shane Elizabeth C. Querubin, Rebecca A. Sukert, Anna Lowien, Alyssa Wellman Houde, Anaïs Roussel, Andrew J. Houston, Ari Cacopardo, Cristy Ho, Haley Talbot-Wendlandt, Jacob M. Widmer, Jairus Slagle, James A. Bader, Jeng Hann Chong, Jenna Wollney, Jordan Kim, Lauren Shepherd, Matthew T. Wilfong, Megan Houlihan, Nathan Sedghi, Rebecca Butcher, Sona Chaudhary, William D. Becker

**Affiliations:** 1 Department of Geology & Earth System Science Interdisciplinary Center, University of Maryland, College Park, Maryland; 2 Pacific Ecological Systems Division, US Environmental Protection Agency, Office of Research and Development, Center for Public Health and Environmental Assessment, Corvallis, Oregon; 3 Cary Institute of Ecosystem Studies, Millbrook, New York; 4 University of Connecticut, Storrs, Connecticut; 5 Occoquan Watershed Monitoring Laboratory, The Charles E. Via Jr Department of Civil and Environmental Engineering, Virginia Tech, Manassas, Virginia;; 6 Center for Coastal Studies, Virginia Tech, Blacksburg, Virginia;; 7 Chatham University, Gibsonia, Pennsylvania;; 8 Department of Environmental Science and Technology, University of Maryland, College Park, Maryland;; 9 Department of Environmental Sciences, University of Virginia, Charlottesville, Virginia;; 10 Environmental Science & Policy Program, University of Maryland, College Park, Maryland;; 11 Department of Biology, Georgetown University, Washington, District of Columbia;; 12 Department of Geology, University of Maryland, College Park, Maryland

## Abstract

Factors driving freshwater salinization syndrome (FSS) influence the severity of impacts and chances for recovery. We hypothesize that spread of FSS across ecosystems is a function of interactions among five state factors: *human activities, geology, flowpaths, climate*, and *time*. (1) *Human activities* drive pulsed or chronic inputs of salt ions and mobilization of chemical contaminants. (2) *Geology* drives rates of erosion, weathering, ion exchange, and acidification-alkalinization. (3) *Flowpaths* drive salinization and contaminant mobilization along hydrologic cycles. (4) *Climate* drives rising water temperatures, salt stress, and evaporative concentration of ions and saltwater intrusion. (5) *Time* influences consequences, thresholds, and potentials for ecosystem recovery. We hypothesize that state factors advance FSS in distinct stages, which eventually contribute to failures in systems-level functions (supporting drinking water, crops, biodiversity, infrastructure, etc.). We present future research directions for protecting freshwaters at risk based on five state factors and stages from diagnosis to prognosis to cure.

## Freshwater salinization syndrome: A growing and diverse problem

Increases in salinity, alkalinity, ionic strength, major ions, hardness, pH, and temperature have been reported over a wide range of freshwaters around the world (Kaushal et al. 2005, 2010, 2018a, 2018b, 2021; Rengasamy 2006; Corsi et al. 2010; Cañedo-Argüelles et al. 2013; Herbert et al. 2015; Dugan et al. 2020; Stets et al. 2020; Thorslund et al. 2021). These increasing salinization trends include freshwaters in North America (Kaushal et al. 2005, 2018b; Dugan et al. 2020; Stets et al. 2020), South America (Kaushal et al. 2021), Asia (Kaushal et al. 2019, 2021), Africa (Kaushal et al. 2021), Europe (Kaushal et al. 2019; Schulz and Cañedo-Argüelles 2019), and Australia (Williams 2001; Kefford et al. 2003; Rengasamy 2006). While freshwater salinization refers to the process of increasing ion concentrations, the complex interrelationships between salt ions, physical, chemical, and biological parameters, and the natural, social, and built environment are called freshwater salinization syndrome (FSS); FSS is characterized by a set of associated consequences or environmental symptoms (Haq et al. 2018; Kaushal et al. 2018b, 2019, 2021; Bhide et al. 2021; Galella et al. 2021). Freshwater salinization only involves increasing salinity in freshwaters. In contrast, FSS involves freshwater salinization and a range of other changes either as a result of the increased salinity or as a result of the factors which caused the increased salinity. Although the presence, magnitude, and extent of the physical and chemical symptoms of FSS varies considerably among watersheds and regions (and there can also be decreasing trends), many major freshwater systems have been impacted globally (Thorslund et al. 2021). Identifying risk factors and diagnosing stages of freshwater degradation due to FSS has significant implications for protecting ecosystem services related to drinking water sources, suitable aquatic habitat, infrastructure, and food/energy production.

**Fig. 1.**
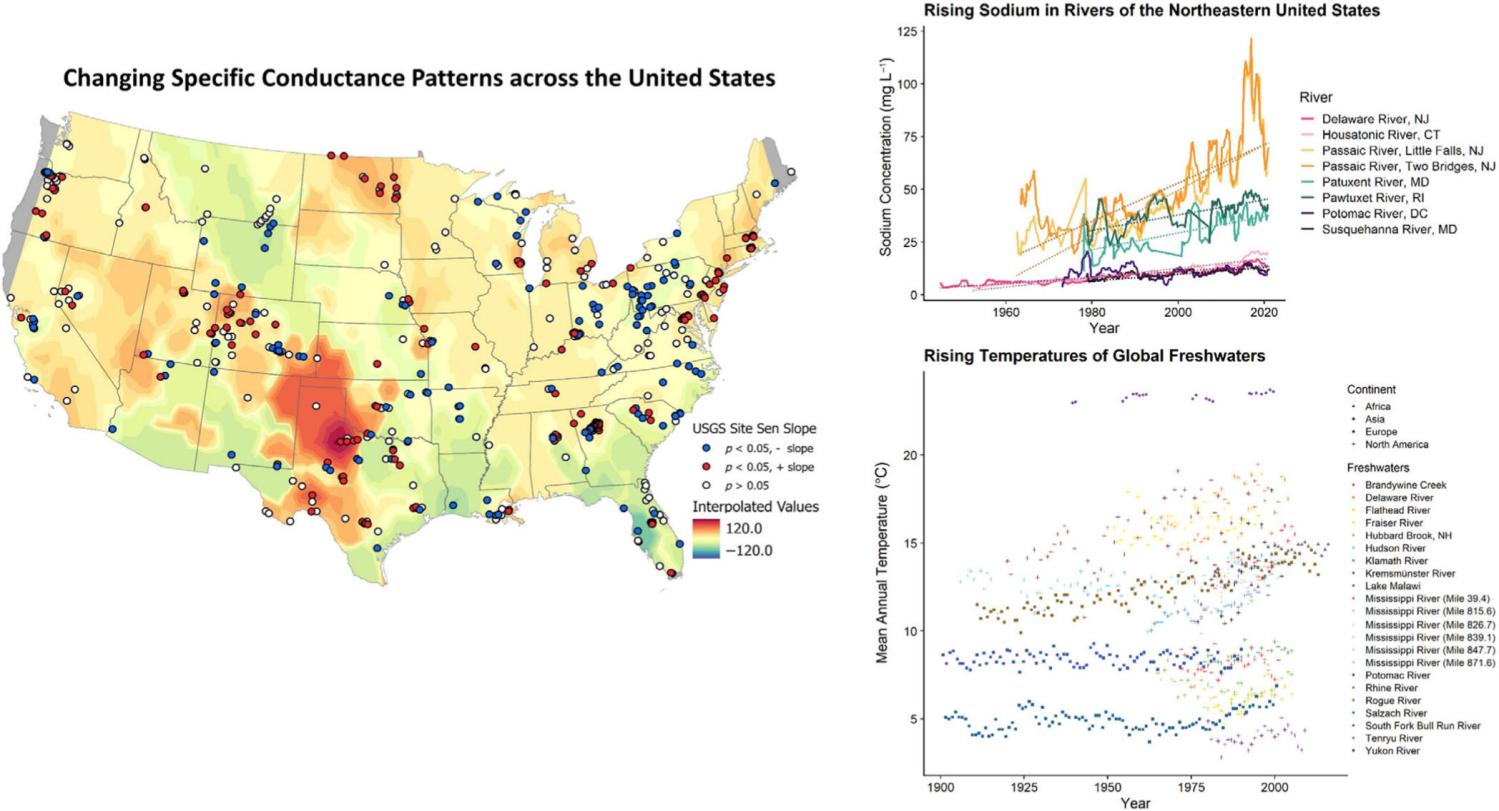
(Left panel) Interpolated values of change in specific conductance (microsiemens per centimeter at 25°C) across streams of the continental United States. Although there are increasing trends, for some sites, specific conductance has decreased or remained unchanged suggesting regional variability. Sen’s slope analyses were performed on data from inland USGS sites with a minimum of a 10-yr span of mean specific conductance recordings. Only sites containing data after 2000 were included. Sites with statistically significant slopes were used to perform ordinary kriging interpolation. Interpolated values were calculated from a spherical semivariogram model (nugget = 0.182, partial sill = 94.8, range = 1.92) in ArcGIS Pro. Warmer colors indicate increasing specific conductance patterns, whereas cooler colors indicate decreasing specific conductance patterns. Areas outside the data extent are represented by gray. The black-outlined dots represent USGS sites. Details on similar analyses can be found in Kaushal et al. (2018b). (Top right panel) Increasing sodium concentrations in rivers of the Northeastern United States; all data are from USGS stream gauge sites. There is not always a continuous increase in Na^+^ concentrations over time and the increase in Na^+^ can sometimes be more apparent at sites, which have higher salinity. Details on similar analyses at sites can be found in Kaushal et al. (2013, 2018b). (Bottom right panel) Increasing temperature trends in global freshwaters. All data are from previously published papers including Kaushal et al. (2010), and references are in Supporting Information.

Although FSS is recognized as an emerging global issue, there can be significant regional variability in responses and symptoms. Major ions have increased over the past century, with most evidence coming from North America, Europe, and Australia (Williams 1987; Kaushal et al. 2005, 2019; Rengasamy 2006; Cañedo-Argüelles 2020; Niedrist et al. 2021). Increases in salt ions reflect a combination of diverse sources, including meteoric salts, water-rock interactions, formation waters (brines in deep groundwaters), anthropogenic salt pollution, and saltwater intrusion (Vengosh 2005; Rengasamy 2006; Herbert et al. 2015; Vengosh et al. 2017; Cañedo-Argüelles 2020; Kaushal et al. 2021). Although some freshwaters show increasing trends in specific conductance on continental and global scales, other freshwaters show that specific conductance has decreased or remained unchanged over time (Fig. 1) (Kaushal et al. 2018a, 2018b; Thorslund et al. 2021). Even within a given region, certain ions like Na^+^ can typically show increasing trends in a majority of rivers, but there can be significant variability in the timing and rates of increase (Fig. 1). In general, while some FSS responses may appear continuous with long-term data in some cases, responses can also be highly pulsed and episodic in some aquatic systems, as revealed by high-frequency sensors (Kaushal et al. 2014b; Haq et al. 2018). Pulses vs. continuous presses of salinization can have differential impacts on ecosystem functions and services over different time scales (Marshall and Bailey 2004; Cañedo-Argüelles et al. 2014). Thus, freshwater salinity is not increasing everywhere and does not always increase in a continuous manner (Fig. 1); however, FSS can develop where salinity is increasing.

Growing evidence for FSS raises potential questions such as: (1) What risk factors influence the development of FSS in some regions and not others? (2) Does FSS progress in stages that can be classified based on chemical and ecological indicators (analogous to trophic states sensu Dodds et al. 1998 or stages of nitrogen saturation sensu Aber et al. 1989)? (3) How do impacts change as FSS stages transition from episodic pulses to chronic long-term presses? and (4) What is the prognosis for recovery for a given waterbody based on its specific risk factors and stages? Based on current evidence, we hypothesize five fundamental variables, or state factors, that can control the progression of FSS on a watershed scale. Although our FSS state factors and stages hypothesis may not be applicable for every world region, it provides an initial framework for hypothesis testing to advance our knowledge regarding the progression of FSS and potential for recovery. State factors have been useful in elucidating the formation of soils (Jenny 1961). Similarly, state factors have been used to explain river chemistry, atmospheric precipitation, rock dominance, and evaporationcrystallization (Gibbs 1970). In general, we hypothesize FSS is a function of interactions among state factors such as: climate, geology, human activities, flowpaths, and time. Identifying state factors and stages of FSS is critical for guiding ecosystem restoration from diagnosis to prognosis to cure.

## A growing need for a state factors and stages approach for addressing FSS

Current evidence shows there are multiple complex biological, chemical, and geological interactions associated with different salt ions. Because of this growing complexity of interactions, new conceptual frameworks are needed to keep pace with organizing information on the growing diversity of drivers and responses. Our state factors hypothesis is a next step following our extensive global review (Kaushal et al. 2021) that conceptualizes how increasing ion concentrations interact with other elemental cycles to influence diverse drivers and progression of FSS. For example, climate has been less explicitly conceptualized in FSS, but this state factor can potentially interact with other state factors to control the rate and geographic spread of FSS. Similarly, the role of natural and anthropogenic flowpaths and interactions with different ions and potential consequences across hydrologic cycles now warrant explicit conceptualization (please see work on irrigation by Thorslund et al. 2021). Because FSS is a systems-level problem, new approaches are necessary to classify and compare state factors among and across ecosystems (Kaushal et al. 2021).

A state factors and stages approach contributes to our understanding of how FSS evolves across space and time and how different state factors interact to influence water quality. State factors and stages can be evaluated as an integrative systems-level conceptual model to categorize risks and multiple impacts across regional scales. State factors can be related to stages, and different state factors may be more important for enhancing certain transitions to progressive FSS stages. For example, climate and geology may play an initial role in early stages that further interact with human activities and flowpaths in later stages over time. Understanding FSS state factors and stages can help better explain ranges in variability in responses across watersheds and regions globally. Ultimately, a state factors and stages approach is a way to collapse the sometimes overwhelming diversity of drivers of FSS and chemical cocktails into understandable categories to advance future research efforts on diagnosing interactive impacts and predicting ecosystem restoration and recovery.

## Part 1. State factors drive progression of diverse forms of FSS

**Fig. 2.**
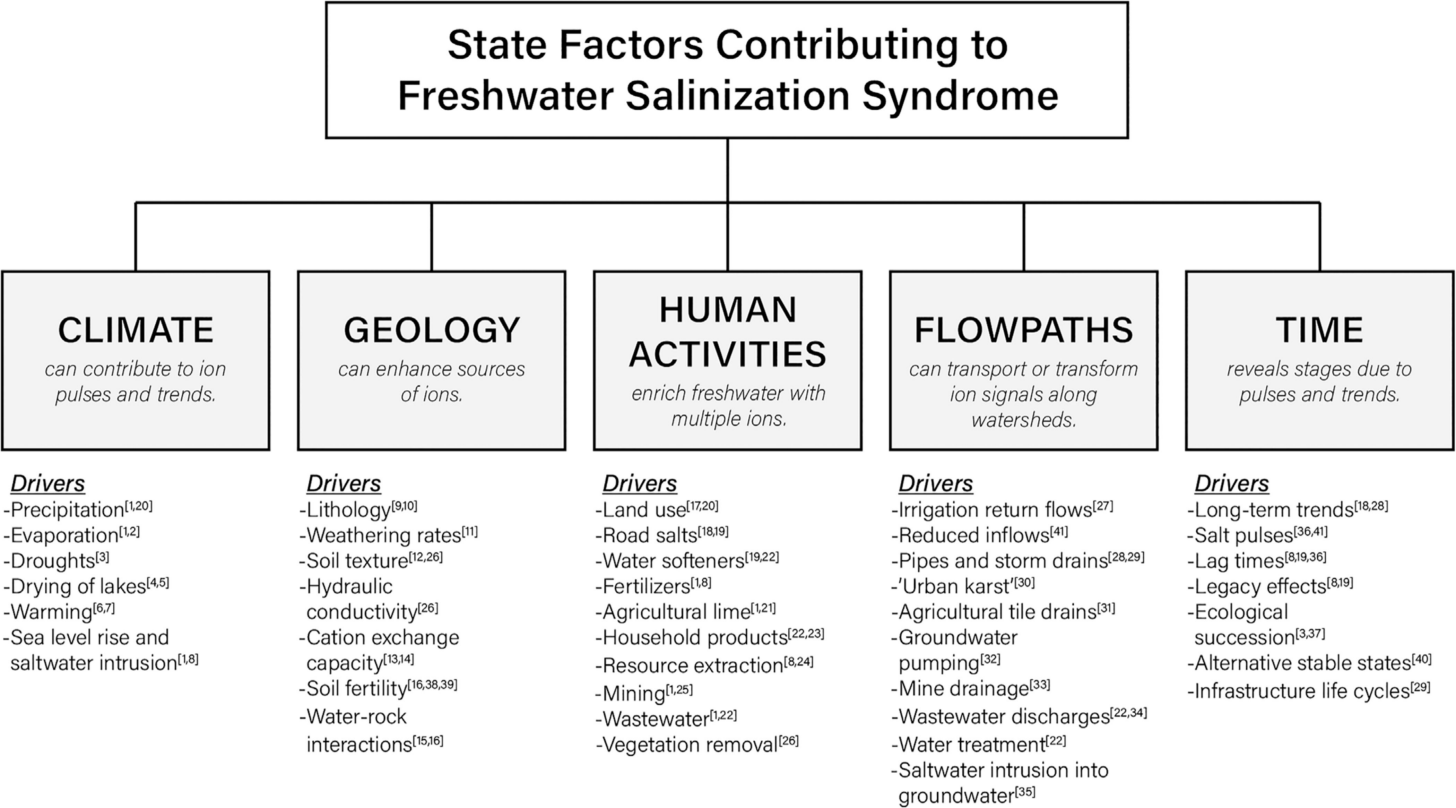
Major state factors influencing the formation rates and progression of FSS. Superscript numbers correspond to literature references found in Supporting Information.

State factors influence the rates of physical, chemical, and biological processes that interactively contribute to FSS and formation of novel and complex chemical mixtures, referred to as “chemical cocktails” hereafter (Kaushal et al. 2018a, 2018b, 2019, 2020, 2021; Morel et al. 2020; Galella et al. 2021). Different regions may show different FSS responses based on climate, geologic and soil properties, vegetation, and topography (Le et al. 2019). We propose that evaluating the relative interactions among state factors can be important in investigating and explaining variability and understanding differences in responses across broader world regions. State factors organize many diverse drivers and related chemical cocktails into categories including urbanization, road salts, agricultural fertilizers, irrigation waters, saline water from aquifers, sea level rise, and saltwater intrusion to name a few (Fig. 2). In this first section, we hypothesize four state factors (human activities, flowpaths, geology, and climate) control the progression and diversity of FSS. We propose time as the 5^th^ state factor in the next section.

### Human activities as a state factor driving progression and diversity of FSS

Human activities interact with all other state factors in driving the spread and diversity of FSS. A few examples associated with interactions between FSS and human activities associated with land development are presented below. Land development is a major human activity related to FSS, but the influence of other human activities on flowpaths, geology, climate, and time are also acknowledged throughout this paper.

## Human development of urban lands drives diverse forms of FSS

Human development of urbanizing lands promotes formation of distinct chemical cocktails through processes like: inputs of road salts (primarily Na^+^ and Cl^−^ but also Ca^2+^, Mg^2+^, etc.), weathering of concrete (Ca^2+^, Mg^2+^, and carbonates), inputs of sewage and animal waste (Na^+^, Cl^−^, and many others), lawn irrigation (Na^+^, Cl^−^), application of lawn fertilizers (N, P, K, and other nutrients), use of water softeners (Na^+^, Cl^−^, K^+^), and alteration of redox reactions due to urban flowpaths and drainage modification (Fe, Mn) (Wright et al. 2011; Kaushal et al. 2018a, 2019, 2020; Morel et al. 2020; Galella et al. 2021). These shifts in major ions influence pH, ion exchange in soils and mobilization and solubility of other metals, ions, and radionuclides (Duan and Kaushal 2015; Haq et al. 2018; Kaushal et al. 2019; Lazur et al. 2020). In colder climates, road salts (deicers) can contribute to large winter pulses in ions and also chronically and steadily increasing baseline concentrations (Kaushal et al. 2005; Dugan et al. 2017; Niedrist et al. 2021; Hintz et al. 2022).

FSS occurs in humid regions (not only those affected by road salts) from human-accelerated weathering of infrastructure in watersheds, sewage leaks (Potter et al. 2014), potable water reuse and irrigation water (Steele and Aitkenhead-Peterson 2011), water softeners, and lawn fertilizers. Depending on climate and underlying geology, urbanization may increase concentrations of various ions including Cl^−^, SO_4_^2−^, Br^−^, Na^+^, Ca^2+^, Mg^2+^, K^+^ (Kaushal et al. 2020). Some of these ions are directly influenced by urban sources whereas others are indirectly influenced by anthropogenically enhanced geochemical processes such as weathering (e.g., Ca^2+^, Mg^2+^, HCO_3_^−^), ion exchange (e.g., Na^+^, Mg^2+^, Ca^2+^, Cl^−^, SO_4_^2−^, NO_3_^−^), and microbial redox reactions (SO_4_^2−^, NO_3_^−^, PO_4_^2−^ bound to Fe and Mn oxides) (Kaushal et al. 2018a, 2020). There may be other processes related to human activities like urban wastewater (Bhide et al. 2021) and unintended consequences of water conservation, which can reduce the amount of water used in homes for household tasks and creates a more concentrated saline wastewater stream (Schwabe et al. 2020) (see section on emerging questions).

## Human development of agricultural lands drives diverse forms of FSS

Human development of agricultural lands enriches freshwaters with multiple ions (Japenga and Harmsen 1990). Nitrate is a highly mobile anion in soils that can promote the leaching of base cations such as Ca^2+^, Mg^2+^, or K^+^. NO_3_^−^ displaces anions (SO_4_^2−^, PO_4_^3−^) and lowers the net negative charge of soil colloids and decreases their capacity to adsorb cations (Davis and Burgoa 1995). Fertilizers that are also enriched with K^+^, Cl^−^, and SO_4_^2−^ can lead to ion pollution (David et al. 2016; Weil and Brady 2016; Hinckley et al. 2020). Gypsum (CaSO_4_) is added to some agricultural soils to promote water infiltration. Additionally, fertilization with ammonium can acidify soils due to nitrification, significantly decreasing soil pH from 5.6 to 4.5 in fields over 5 yr (Juo et al. 1995) and increase soil weathering rates. Additions of agricultural lime to counteract acidification further increase major ions.

### Flowpaths as a state factor driving diversity and progression of FSS

Natural and engineered flowpaths warrant special recognition because they determine how far and fast salt ions from diverse sources travel downstream. Flowpaths integrate across the four other state factors—climate, geology, human activities, and time—by connecting ion sources released at various locations and times in a watershed to sensitive downstream water bodies. Flowpaths also encapsulate the size of drainage networks and catchments, which can influence vulnerability to FSS in some cases. Whether flowpaths lead to progressive enrichment or dilution of salt ions and/or mobilization or attenuation of secondary chemical cocktails along drainage networks is important to consider when predicting degradation vs. recovery.

### Irrigation flowpaths drive FSS

Agriculture contributes to diverse forms of FSS by modifying hydrologic flowpaths associated with irrigation cycles and return flows. Irrigation has recently been shown to be an important contributor to salinization across regions (Thorslund et al. 2021). We include irrigation under the flowpaths state factor because it is a process involving the transport and flow of water and solutes, and is an important component of the hydrologic cycle in human-dominated landscapes (Shibuo et al. 2007) (Fig. 2). Agricultural soils in arid regions and poorly drained soils can accumulate ions at the soil surface as a salt crust. Salt residues remain from evapotranspiration when crops use irrigation water (Vengosh 2005). As groundwater becomes saline, and is used over again as irrigation water, a perpetual cycle of increasing salinization develops in agricultural systems (Weil and Brady 2016).

### Vegetation and soil disturbance drives FSS along groundwater flowpaths

Disturbance of plant communities, through practices like deforestation and clear-cutting, enhances salinization by altering the hydrological and nutrient cycles. Plants take up many ions such as Ca^2+^, K^+^, Mg^2+^, Na^+^, NO_3_^−^, PO_4_^3−^, SO_4_^2^, Cl^−^, etc., which originate from weathering of soil minerals, atmospheric deposition, and/or decomposition of organic matter (Likens et al. 1970, 2021; Likens 2013). When trees are cut, major ion concentrations increase because plant uptake of ions from soil and groundwater resources are decreased, while erosion and inputs and mineralization of dead and decomposing organic matter increases (Likens 2013). In addition, vegetation can regulate groundwater salinity by influencing evapotranspiration and vertical flowpaths of ions along soil profiles (Si, K^+^, Ca^2+^, Mg^2+^, and HCO_3_^−^) (Vengosh 2005; Humphries et al. 2011).

### Groundwater pumping and resource extraction drives FSS along flowpaths

Extraction of water, minerals, and energy for resources along flowpaths further contributes to diverse causes and consequences of FSS. In some regions, over pumping of groundwater contributes to salinization, particularly in coastal areas due to saltwater intrusion and displacement of fresh groundwater (Glazer and Likens 2012; Tully et al. 2019a) (Fig. 2). Mining and oil and gas production release salt ions along flowpaths by exposing fresh surficial rocks and bedrock to weathering processes (Palmer et al. 2010; Bernhardt and Palmer 2011; Vengosh et al. 2017). During oil and gas production, groundwater brines may be released into the surface environment along flowpaths. These deep groundwater brines can be mixtures of ancient trapped seawater and/or groundwater with ion chemistry heavily modified by rock–water interactions (Vengosh 2005). In other cases, fluids enriched in solutes are injected into the ground for gas extraction through hydraulic fracturing (Vengosh et al. 2017). Diverse types and effects of salinization processes associated with mining and resource extraction have been reviewed elsewhere (Bernhardt and Palmer 2011; Cañedo-Argüelles et al. 2017; CañedoArgüelles 2020; Kaushal et al. 2021).

## Diverse flowpaths through catchments and legacy pollution sources drive FSS

Once released, salt ions move along a diverse array of flowpaths through a catchment, with varying transit times (i.e., the time it takes the water and associated salt ion to travel from its point of origin in the catchment to a stream or other receiving water of interest) (Kirchner et al. 2000; Hrachowitz et al. 2016). This diversity of flowpaths and transit times implies that the introduction of salt ions to a catchment (e.g., as fertilizers in agriculture settings or road salt along highways, see above) can enter sensitive receiving waters over timescales ranging from minutes up to decades or longer, in extreme cases like nitrate in the Mississippi River Basin (even after all sources have been removed) (Van Meter et al. 2018) (for more details, please see section “Modeling diverse flowpaths through catchments and legacy pollution sources” in Part 3. Future research directions and knowledge gaps).

### Geology as a state factor driving diversity and progression of FSS

#### Human-accelerated weathering due to acidic precipitation drives FSS

Acidic precipitation is a major driver of human-accelerated weathering of ions. Acid rain can deplete soil sources of base cations and increase leaching of base cations to streams; however, the impacts of acid rain on FSS are strongly dependent on the susceptibility of lithology to weathering. For example, resupply of base cations may be limited in regions where the lithology is dominated by less-weatherable materials, such as igneous and metamorphic bedrock (Likens et al. 1996; Borg and Sundbom 2014) and in base cation-poor soils with crystalline lithology that are less susceptible to weathering (Likens et al. 1996; Likens and Buso 2012; Likens 2013). In contrast, regions dominated by calcareous bedrock or other easily weathered sedimentary rock can increase acid neutralizing capacity and river alkalinization by replenishing base cations and carbonates to surface waters (Kaushal et al. 2013). Thus, the resulting effects and progression of FSS from early to more advanced stages is dependent on soil buffering capacity, as well as the length of time soils are exposed to acid rain sources.

#### Human-accelerated weathering from mining drives FSS

Much work has synthesized and reviewed the impacts of acidification and mine drainage on water quality on a global scale (Rice and Herman 2012), and we will only highlight its connection to FSS as a driver, which has been less emphasized. Mine drainage accelerates weathering of rocks and soils increasing concentrations and fluxes of major ions and trace elements to groundwater and surface waters. Mine drainage can lead to increased river acidification or alkalinization, specific conductance, and major ion concentrations (Bernhardt and Palmer 2011). Mining has diverse consequences on FSS across world regions. For example, potash mines in Germany and Spain have contributed to freshwater salinization and can increase toxicity to organisms for K^+^ ions (Gorostiza and Saurí 2019; Schulz and Cañedo-Argüelles 2019).

#### Human-accelerated weathering of construction materials drives FSS

Concrete, asphalt, and other construction materials influence the magnitude and diversity of FSS and major ions (Wright et al. 2011) (Fig. 2). These materials are more susceptible to weathering based on their mineral composition. Although many geologic materials used in construction contribute to FSS, we focus here on concrete due to its global importance. Concrete, in use since as early as 6500 B.C.E. (Sahafnia 2018; Toma et al. 2020), is a proportioned mixture of cement, water, and fine and coarse aggregate. Concrete is weathered physically and chemically by many of the same processes that affect natural lithology; freeze/thaw cycles, abrasion, and thermal expansion/contraction cycles are especially important weathering processes.

### Climate as a state factor driving diversity and progression of FSS

Freshwater salinization is especially common in semi-arid and Mediterranean climates, where humidity is generally low. Rising temperatures can increase ion concentrations through increased evaporation and evapotranspiration (Marie and Vengosh 2001; Salameh 2001) (Fig. 2). Rising temperatures also accelerate watershed weathering rates (Raymond 2017). In addition, climate variability enhances oxidation of sulfide minerals during droughts and increases SO_4_^2−^ concentrations during re-wetting events (Simpson et al. 2010; Kaushal et al. 2018a), which may also influence weathering rates. However, other work forecasts a relatively modest rise in salinity due to climate change (10–15%) (Le et al. 2019).

Sea level rise and saltwater intrusion are major drivers of FSS in coastal and estuarine environments, increasing mixing of marine or brackish water into surface and groundwater resources (Barlow and Reichard 2010; Carr et al. 2020). Marine seawater composition varies somewhat around the globe but it is a well-defined chemical mixture, and its dominant ions in decreasing molar concentration are: Cl^−^, Na^+^, Mg^2+^, SO_4_^2−^, Ca^2+^, and K^+^ (Millero et al. 2008). Several factors drive saltwater intrusion including increased storm surges, relative sea level rise, failing drainage infrastructure, and groundwater pumping (Ketabchi et al. 2016). Storm surges and higher tides can bring salt water into direct contact with freshwater as land is periodically or permanently inundated (Tully et al. 2019a). Freshwater salinization can also increase when flow reverses in drainage networks such as ditches or storm drains, allowing salt water to directly flow deep into once drained landscapes and even to pass vertically between aquifers through wells and boreholes (Barlow and Reichard 2010).

## Part 2. Time as a state factor: Progression of FSS stages and prognosis

**Fig. 3.**
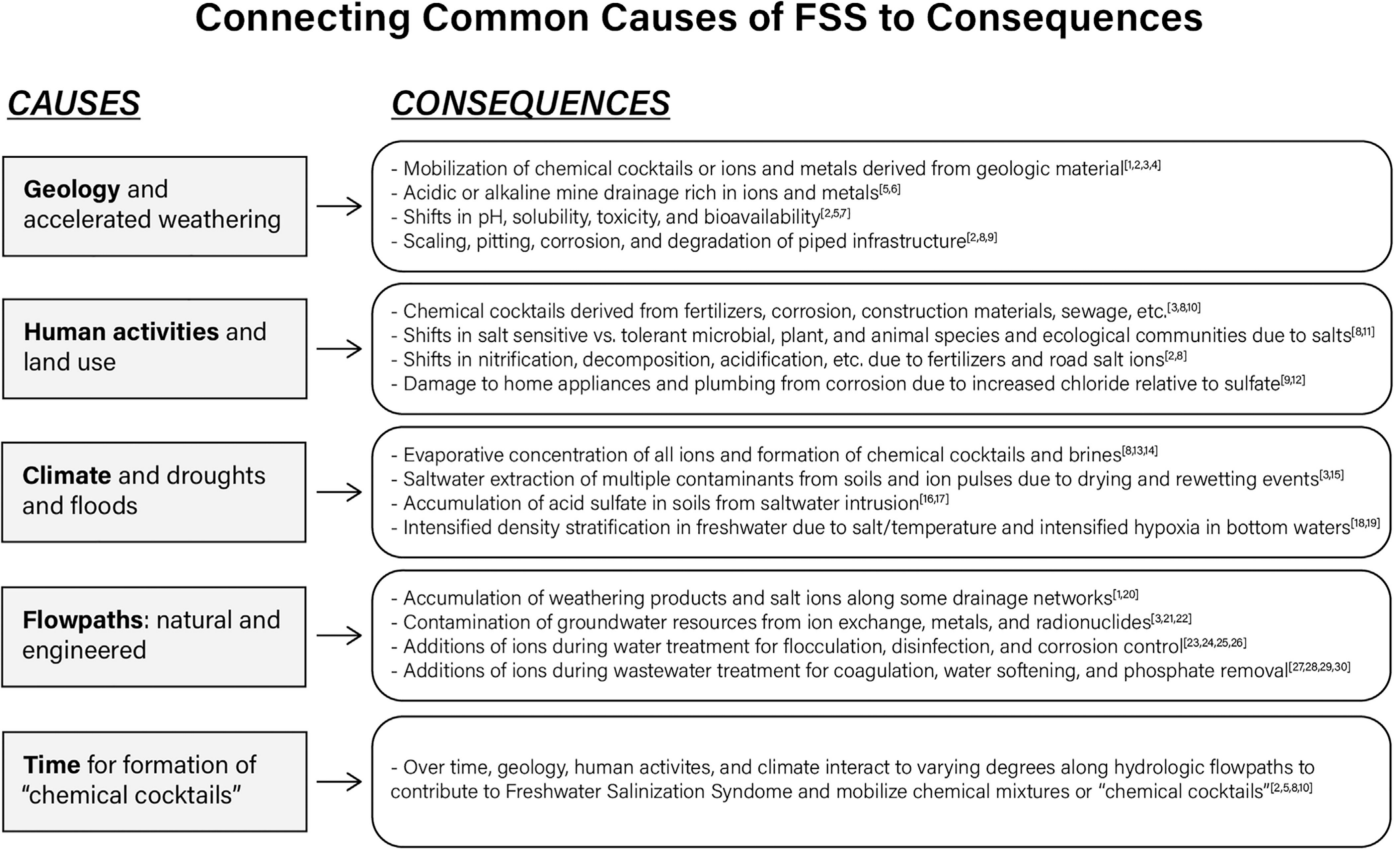
Common causes and consequences of FSS are often connected. Superscript numbers correspond to literature references found in Supporting Information.

**Fig. 4.**
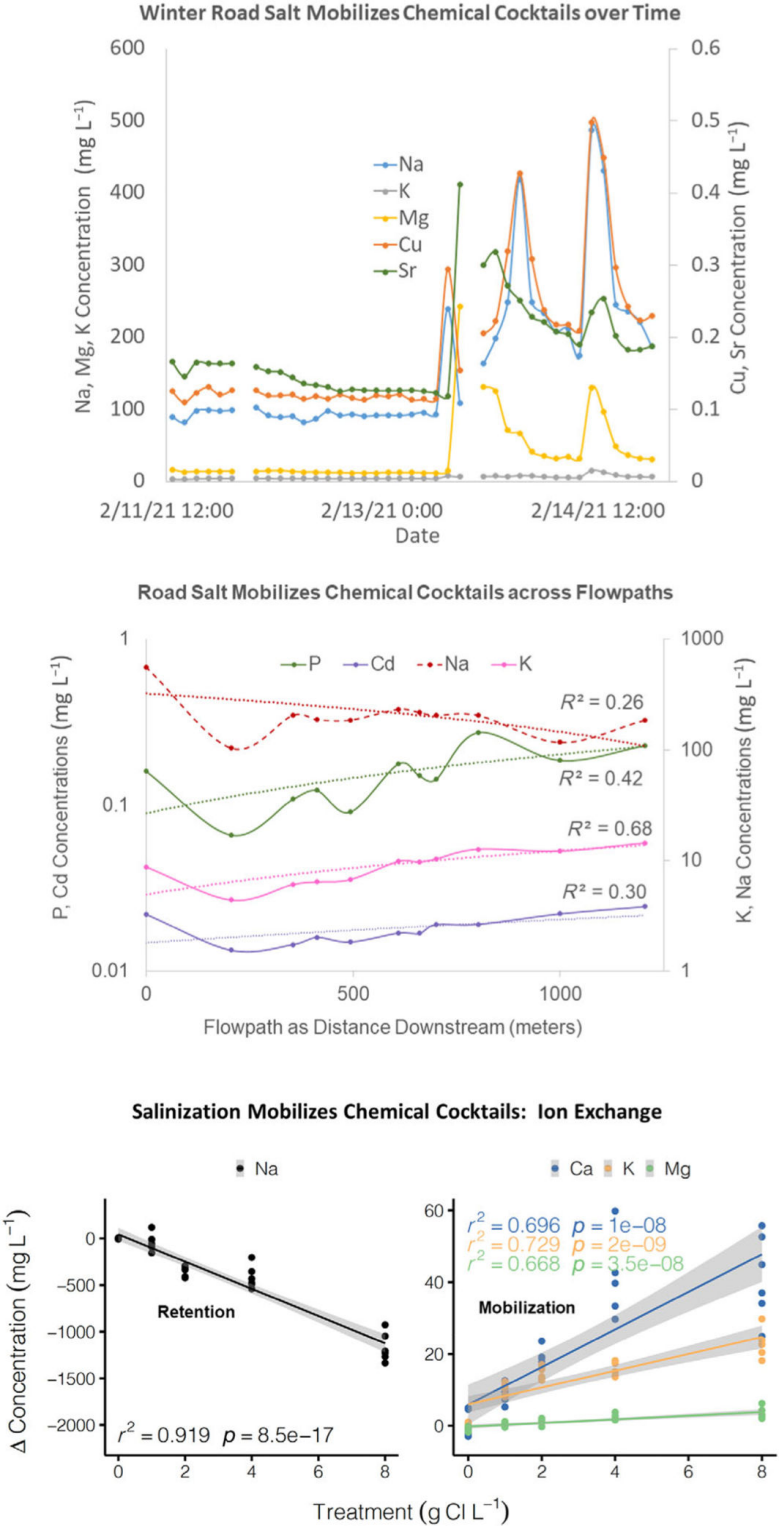
FSS mobilizes chemical cocktails across space and time in Campus Creek, a small urban stream in College Park, Maryland. Campus Creek has undergone a type of stream restoration known as regenerative stormwater conveyance, which dramatically enhances floodplain reconnection through the creation of a series of step pools. (Top panel) In Campus Creek, there are peaks in ion concentrations during a winter road salt event illustrating the importance of mobilization of multiple elements over time in response to deicer applications and winter climate. (Middle panel) Along the longitudinal flowpath of Campus Creek, Na concentrations decline during winter after a road salt event, as other elemental concentrations increase (all slopes are significantly different than zero); this suggests the importance of ion exchange and/or shifts in sources along the flowpath. (Bottom panel) Laboratory salinization experiments with sediments from Campus Creek demonstrates the importance of Na retention as NaCl is added at increasing concentrations (using experimental methods similar to Haq et al. 2018 and Kaushal et al. 2019); other elements are mobilized and released from sediments of Campus Creek due to ion exchange and geochemical processes.

Depending on human activities, climate, geology, and flowpaths, FSS can evolve over time through recognizable and generalizable stages. In the early stages, we hypothesize that climate and geology influence background risk factors for developing FSS, but the interaction of human activities along flowpaths can rapidly accelerate transitions to advanced stages of FSS. FSS stages can be characterized by ranges and shifts in ion mobility, ion exchange capacity of soils, biological toxicity, persistence and transformation in the environment, etc. (Table 1; Figs. 3, 4). These shifts in ecosystem processes, functions, and services can manifest as changes in the formation of chemical cocktails and ecosystem impacts across later stages of FSS within watersheds (Fig. 4).

**Fig. 5.**
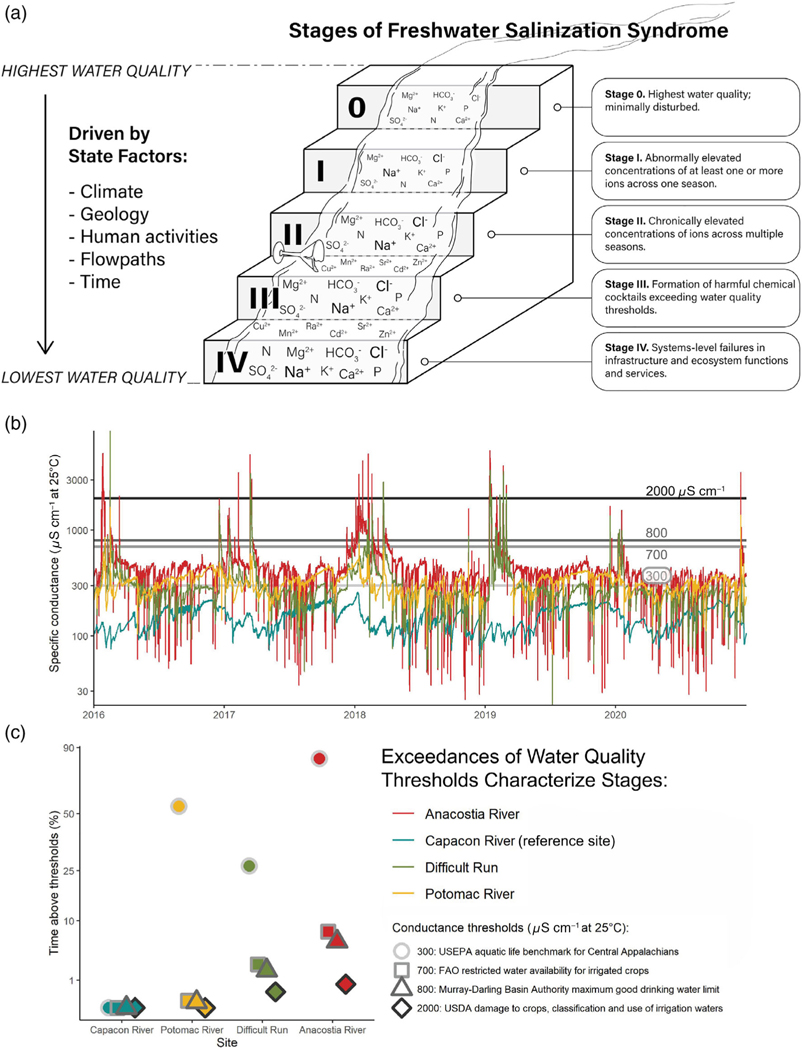
(Panel a) There can be a progression of FSS through distinct stages that can be diagnosed with water quality and other data. Based on state factors, diverse consequences of FSS can evolve across stages characterized by shifts in ion mobility, ion exchange capacity of soils, biological toxicity, persistence and transformation in the environment, etc. (Panel b) Specific conductance data recorded at high temporal resolution in four lotic systems of the mid-Atlantic U.S. Data collected from automated specific conductance records in the Anacostia, Capacon, and Potomac Rivers and Difficult Run from 2016 to 2021. The Capacon River is a minimally disturbed reference site and the Anacostia and Potomac Rivers and Difficult Run are affected by urbanization, agriculture, and other impacted land uses. In situ readings were recorded every 5 or 15 min at each site. (Panel c) The percentage of time each of the four waterways spent above three key thresholds over the 5-yr period depicted above. Thresholds for conductivity are derived from U.S. and Australian federal water quality management agencies and the United Nations Food and Agricultural Organization.

As a starting point to catalyze research, we hypothesize a progression of FSS through stages at a watershed scale that can be investigated as follows (Fig. 5): Stage 1 is characterized by abnormally elevated concentrations of ions during at least one season; Stage 2 is characterized by chronically elevated concentrations of ions throughout multiple seasons; Stage 3 is characterized by the formation of complex, distinct, and harmful chemical cocktails near thresholds for water quality; and Stage 4 is characterized by chronically elevated concentrations of ions and progressive secondary mobilization of chemical cocktails, which eventually contribute to failures in systems-level services (supporting drinking water, biodiversity, and infrastructure) in the watershed. It is important to hypothesize that a given waterbody can experience these stages over varying durations throughout the year, and it may only be a certain proportion of the time (Fig. 5). In some cases, smaller watersheds may be more at risk from FSS than larger watersheds and spend a greater proportion of time in advanced stages of FSS. Below, we provide a few examples of what may occur across hypothesized stages of FSS.

### Hypothesized stage 1 of FSS (seasonal pulses and short-term recovery)

Increasing seasonal salt concentrations in freshwaters from human activities cause immediate osmotic stress to biotic communities. Species adapted to life in freshwaters are not always tolerant to high salt concentrations (Ury et al. 2019); however, many species can overcome short-term osmotic imbalances due to salt pulses over time. As an example, some organisms can use organic and inorganic solutes to balance internal salt concentrations in cells (Griffith 2017). Flowpaths that result in salt intrusion are swift, and abrupt shifts in environmental conditions can sometimes influence biomass, behavior, and/or biological activity of certain organisms, under certain circumstances (Hintz and Relyea 2017). Episodic events may not result in noticeable or even measurable changes in systems with high buffering capacity or may lead to significant detectable changes in some cases (Oliveira et al. 2021). As episodic events become more frequent over time, and salt concentrations increase, ecological communities should eventually shift to more salt-tolerant species across progressive stages of FSS (below) (Ury et al. 2019).

### Hypothesized stage 2 of FSS (chronically elevated ions across seasons)

Chronically high salt concentrations cause molar imbalances resulting in base cations and hydrogen (H^+^) ions bound to soil colloids to be displaced likely by Na^+^ (Haq et al. 2018) (Table 1). Base cations are lost as soil water moves downstream along flowpaths, and there are temporary shifts in soil pH and episodic acidification (Green and Cresser 2008) (Table 1). After episodic pulsed events, base cations are replenished based on underlying geology and weathering. Depending on climate and flowpaths, Na^+^ ions can still be displaced from the soil colloids and flushed from the system (Table 1); soil-buffering capacity can then be restored over time. When soils have a refractory geology (i.e., quartz, muscovite, etc.), soil pH can become more alkaline and recovery from episodic events may be delayed over time. In systems with little base cation rejuvenation and flushing, high Na^+^ levels will concentrate and weaken soil structure over time.

### Hypothesized stage 3 of FSS (mobilization of harmful chemical cocktails)

In this stage, FSS degrades soil structure, which negatively affects plant root penetration, soil water conductivity, nutrient retention, and other critical ecosystem functions and services (Rengasamy 2006) (Table 1). Suspension of soil particles also increases sediment transport and mobilizes chemical cocktails of dissolved organic matter and colloid bound trace metals (Table 1). Formation of harmful chemical cocktails can occur as seasonal pulses or as water travels along flowpaths due to ion exchange and other biogeochemical processes (Fig. 4). Long-term salinization leads to widespread mobilization of multiple, harmful chemical cocktails, nutrient deficiencies, and altered biogeochemical cycling (Duan and Kaushal 2015; Kaushal et al. 2019) (Table 1). For example, high concentrations and repeated applications of road salts can disrupt and mobilize organic matter and dissolved organic nitrogen (Green et al. 2008a,b, 2009) (Table 1). This mobilization of organic matter can increase decomposition rates and overall pore-water NH_4_^+^ (i.e., ammonification). Long-term salt additions can also sometimes raise overall pH values (Green and Cresser 2008), which lead to higher rates of ammonium oxidation (i.e., nitrification) (Green and Cresser 2008) (Table 1). Although chemical cocktails form at lower concentrations of salinity (earlier stages of FSS), concentrations and compositions of chemical cocktails have now begun to approach and/or exceed thresholds for water quality.

### Hypothesized stage 4 of FSS (systems-level failures from salinization)

This stage of the FSS is characterized by the failure of system-level functions and services, including crop production, potable drinking water, biodiversity infrastructure, ecosystems, and aquatic and human health. Examples include: (1) increases in chloride to sulfate mass ratios triggering corrosion in pipes and contamination of drinking water tainted with heavy metals (Edwards and Triantafyllidou 2007); (2) mobilization of nutrients, radionuclides, and other contaminants with potential impacts on aquatic and human health; (3) alternative stable states and trophic cascades due to meromixis in lakes or food web alterations; (4) crop death and losses in soil fertility. Further discussion of emerging risks across multiple systems can be found in Kaushal et al. (2021) and references throughout this paper.

Future research may investigate applications of these FSS stages for monitoring and diagnosing the underlying drivers, identifying mitigation strategies and predicting recovery. Possible questions surrounding the practical application of FSS stages on a broader regional scale include: (1) Does stage 0 require a certain proportion of the sites to be of the highest water quality? (2) Is there a threshold of sites within a region past which a region transitions from one stage to another? (3) How should a region be defined when evaluating a stage—by climate, geology, flowpaths, human activities, or other state factors? (4) What is the temporal duration and frequency necessary to monitor and diagnose a freshwater body or region for FSS? and (5) Does the spatial extent of these stages increase with increasing stage? What causes FSS to spread through space might be different from what determines the background vulnerability of a particular water body to elevated base cations and anions. Answers to these questions will involve further analysis and a holistic identification of the progression of symptoms.

### Considering progression of FSS in biota and ecosystems

The biological consequences of FSS have been documented once salinity passes certain thresholds in soils and aquatic environments adjacent to and downstream from roadways. As an example peak values of chloride in suburban and urban streams experiencing FSS can be between 30 and 200 mg L^−1^ in summer and between 181 and 4600 mg L^−1^ in winter (Kaushal et al. 2005). These stream chloride ion concentrations are well within the “moderately saline” classification used by the US Geological Survey (USGS) and, in winter months exceed aquatic chronic toxicity thresholds (Environment Canada 2001). The USGS defines freshwater as <1000 mg L^−1^ dissolved salts (Winslow and Kister 1956).

Salinity can be a strong driver of microbial community composition and biogeochemical functions and processes as well, but there are many unknowns regarding thresholds and biological regime shifts across stages of FSS (Yue et al. 2019). For example, understanding how microbial communities adapt to salinity stress over time is key to understanding how ecosystems will respond to global change across stages of FSS (Yan and Marschner 2012). Increased salinity impacts nutrient cycling and decomposition rates (Green et al. 2008a,b, 2009; Duan and Kaushal 2015; Haq et al. 2018). Variability in salt tolerances and thresholds among aquatic organisms also can affect food webs indirectly (Cañedo-Argüelles et al. 2016; Hintz et al. 2017). Mesocosm experiments with multiple producers and consumers found that zooplankton are less salt tolerant than frog (*Hyla versicolor*) tadpoles, phytoplankton, and periphyton (Van Meter et al. 2011). Increased algal abundance due to decreased herbivory allowed tadpoles to grow larger and metamorphose faster in saline waters than in the control group (Van Meter et al. 2011). Salinization can also induce cascade effects through food webs (Moffett et al. in press). More work is necessary using ecosystem level experiments to understand changes in ecosystem functions and services across stages (Cunillera-Montcusí et al. 2022).

### Considering progression of FSS from interactions with geology

The consequences of acidification–alkalinization on major ion concentrations and chemistry have been extensively reviewed (Likens et al. 1979; Haines 1981; Rice and Herman 2012; Kaushal et al. 2013, 2017; Likens 2013). Depending on the concentrations of acid ions, the pH of surface waters may be lethal to many macroinvertebrates and fish species (Haines 1981); lower surface water pH can mobilize aluminum (Al), resulting in Al levels that are lethal to many macroinvertebrate and fish species. Additionally, decreased pH of surface waters allows for the release, mobilization, and increased bioavailability of potentially toxic metals such as Cd, Cu, Pb, and Zn (Sandifer and Hopkin 1996) (Table 1; Fig. 3). Freshwaters with increased salinity can also result in the mobilization of heavy metals along hydrologic flowpaths particularly via complexation with Cl^−^ and SO_4_^2−^. A decrease in surface water pH and increase in bioavailable metal concentrations may have additive or interactive effects on toxicity for aquatic biota especially in headwaters and urban areas (Komínková et al. 2016). Conversely, the alkalinization of surface waters and increase in base cations, pH, and water hardness can increase primary productivity (DIC and K^+^), shell thickness (increased calcite saturation to form shells), and alter food webs (Kaushal et al. 2013; Strayer et al. 2021). Increased Ca^2+^ and Mg^2+^ can also alter the bioavailability of some toxic metals by outcompeting metal ions for binding onto biotic ligands reducing toxicological effects (Paquin et al. 2002).

### Considering progression of FSS from interactions with climate

There are many questions regarding how climate change will impact salt use, management, and system-level processes and interactions (Fig. 3). For example, there could be less use of road salt due to global warming in regions where frozen precipitation is predicted to decline under most climate scenarios. On the other hand, droughts, evaporation, and sea level rise can increase salinization (Jeppesen et al. 2020). Mineralization and increase of N, P, and C concentrations in drainage waters have shown a positive correlation with increasing air/water temperatures and/or wetting/drying cycles (Kaushal et al. 2008, 2014a; Lepistö et al. 2008; Delpla et al. 2009). Saltwater intrusion also leads to pulses of N and P concentrations due to salt extractions of nutrients from agricultural soils from ion exchange and biogeochemical processes (Ardón et al. 2013; Tully et al. 2019b) (saltwater intrusion is discussed further below). Below are a few general considerations for how FSS could progress via interactions with climate.

## Interaction between FSS and temperature can amplify stratification and hypoxia

Density stratification of lakes, estuaries, and rivers is enhanced by increasing air temperature (e.g., influenced by climate change) and also by increased salinization impacts (Novotny and Stefan 2012; Sibert et al. 2015) (Fig. 3). Increased temperature and salinity decreases O_2_ solubility and leads to the release of P in anoxic bottom sediments (Delpla et al. 2009). Diurnal stratification in the water column is also likely to be amplified, which also may lead to episodic anoxic environments and release of P and metals from sediments in reducing environments with potential shifts in ion exchange (Giles et al. 2016; Ladwig et al. in press). Increasing water temperatures enhance rates of CO_2_ and CH_4_ pulses due to higher ecosystem respiration (Kaushal et al. 2014a), but interactive effects of different salt ions such as SO_4_^2−^ on microbial processes and trace gases in bottom sediments warrant further research (Helton et al. 2014).

## Interaction between FSS and climate can alter species diversity and abundance

The salinity and temperature of water regulate biological, toxicological, and ecological processes, leading to interactive effects on species diversity and abundance. Changes in salinity and temperature can increase the abundance and distribution of tolerant taxa while decreasing sensitive species. There can be interactions between temperature and salinity, where ion toxicity is greater at warmer temperatures (Jackson and Funk 2019). For example, there is increased toxicity from SO_4_^2−^ to mayflies and osmoregulation can be disrupted by elevated salinities and water temperatures (Orr and Buchwalter 2020). Temperature, salinity, and dissolved oxygen can interact as multiple stressors to influence aquatic insects and their modes of respiration (Verberk et al. 2020). In addition, salinity and temperature can interact to increase the toxicity of other contaminants such as herbicides and heavy metals (Mclusky et al. 1986; Gama-Flores et al. 2005; DeLorenzo et al. 2013). Some changes in species could be related to changes in the survival of pathogens and harmful algal blooms in freshwaters influenced by salinization and warming. DeVilbiss et al. (2021) showed that salinization increases *Escherichia coli* survival rates, with Mg^2+^ and Cl^−^ concentrations having differential effects on survival. Other work has shown that freshwater salinization can mobilize nutrients (Steinmuller and Chambers 2018), which could affect algal blooms. More work is necessary to investigate the potential interactive effects of salinization and warming on changes in species diversity, abundance, and survival in freshwaters.

### Considering progression of FSS from interactions along flowpaths

The consideration of flowpaths as a state factor of FSS is new, and it has broad significance for the progression of FSS in many inland waters and engineered systems. For example, reduced surface flow in tributaries along flowpaths can reduce lake water levels and contribute to FSS (the Aral Sea is a very good example of that). In addition, flowpaths associated with irrigation can contribute to FSS (as mentioned earlier). Many examples considering progression of FSS along flowpaths can be found in Fig. 2. Below, we focus on saltwater intrusion as just one example of the importance of recognition of flowpaths as a state factor for driving FSS. We further discuss the importance of flowpaths in the section below regarding future research.

Saltwater intrusion leads to consequences that impact land use, ecology, geomorphology, and even the mineralogy of soils and geologic strata (Fig. 3). Initially, as coastal inundation is intermittently increased by storm surges and the highest tides, salt intolerant plants die back but leave a barren salted edge where salt tolerant vegetation has not yet colonized (Tully et al. 2019a). As land and freshwater are salinized, marine (or more dilute brackish) waters can interact with soils and sediments to extract bound ions, particularly N and P in agricultural landscapes, creating new chemical cocktails that are enriched in nutrients and trigger algal blooms in surface waters (Kristensen et al. 2021). Ecological and land use changes extend beyond replacement with salt tolerant plant species, as agricultural land is completely abandoned and forests consisting of salt intolerant tree species die back to form ghost forests of dead trees standing in recently migrated salt marshes (Kirwan and Gedan 2019; Ury et al. 2021). Geomorphologic changes can occur quickly after these ecological changes; in the case of ghost forests, the large woody roots rot following tree death and can cause the land surface to collapse (Carr et al. 2020). Inundated mineral material itself begins to change as metal oxides are reduced and new forms precipitated (Tully et al. 2019b) and the SO_4_^2−^ in seawater is reduced via microbial activity to form sulfide minerals such as pyrite (Wessel and Rabenhorst 2017).

## Part 3. Future research directions and knowledge gaps

The FSS state factors and stages hypothesis condenses diverse drivers, interactions, and chemical cocktails into understandable categories to advance future research. Below, we present a few key research directions with an emphasis on diagnosing interactive impacts along natural and engineered flowpaths, salt use at the intersection of built and natural systems, and elucidating legacy impacts of salt ions along flowpaths.

### How does FSS evolve along natural and engineered flowpaths?

The FSS is amplified as water circulates through the critical engineering infrastructure and flowpaths needed to sustain the water supply and waste disposal needs of urban and periurban populations (Bhide et al. 2021). Salts are added at multiple points in water and wastewater systems including: (1) at the drinking water treatment plant (Na^+^, Al^3+^, Fe^3+^, Fe^2+^, CO_3_^2−^, Cl^−^, F^−^, PO_4_^3−^) for flocculation, disinfection, and corrosion control (Letterman and AWW Association 1999; Wiest et al. 2011; Shammas and Wang 2015); (2) as a by-product of industrial, residential, and commercial activities, leading to the discharge of diverse salt loads to the sewage collection system (e.g., Na^+^, F^−^, Cl^−^, Mo, Cr, PO_4_^3−^) discussed further below; and (3) at the wastewater treatment plant (Na^+^, Ca^2+^, Fe^2+^, Fe^3+^, Al^3+^, Cl^−^, SO_4_^2^) for coagulation, water softening, phosphate removal, disinfection, de-chlorination, and odor control processes (Lazarova et al. 1999; USEPA EPA 2000; Davis 2010). Because salts are generally not removed by conventional wastewater practices, these added ions pass directly through wastewater treatment plants to receiving waters, thereby contributing to the FSS. Treated wastewater discharged to a stream becomes source water for drinking water treatment plants downstream, resulting in a downstream “spiraling” and progressive addition and concentration of ions and other pollutants (e.g., pharmaceutical compounds) between the stream and adjacent communities reliant on the stream for their water supply and wastewater removal (Fork et al. 2021) Indeed, > 50% of the flow in 900 streams in the United States consists of wastewater effluent (Rice and Jastram 2015). Ironically, efforts to reduce water consumption in urban settings, like Southern California, can result in more concentrated sewage with higher ion concentrations, and higher total dissolved solids in wastewater effluents (Schwabe et al. 2020). There is also the potential for evaporative concentration of ions by system-scale recirculation of water and associated salts, as for example, with the reuse of treated wastewater to satisfy commercial and industrial demand for nonpotable water.

The many sources of salt ions added to urban water systems create opportunities to reduce salt loads (mass per time) discharged to receiving waters, and thus the FSS, over a range of scales and across businesses, industry, government, and individual homeowners. One example is social marketing interventions aimed at encouraging product switching at the household scale, to reduce ion loads discharged to the sewer system and, ultimately, to receiving waters. The most comprehensive evaluation of the ion content of home products was conducted by the Commonwealth Scientific and Industrial Research Organization during the Millennium Drought in Melbourne Australia (Tjandraatmadja et al. 2010). The study evaluated 156 household products and identified ion fingerprints associated with different product types; for example, F^−^, Cl^−^, Mo, Cr, and total P were found to co-occur in laundry powder, toilet fresheners, and dishwasher powder, but not in fabric softener or sunscreen. Some 80% of all products tested contained detectable levels of Cl^−^, with powdered laundry detergent having the highest weekly Cl^−^ mass loading to the sewer (on the same order of magnitude expected from human excreta; Vinnerås 2001; Tjandraatmadja et al. 2010). Ion loading varies dramatically across household products and brands; in particular, switching to “environmentally friendly” products decreased Cl^−^ loading to the sewer system by 24–98% (Tjandraatmadja et al. 2010). More research is needed to evaluate the effectiveness of product switching in practice, and more generally, the efficacy of social marketing efforts to reduce salt loading to the sewershed. Such interventions can be framed as an attempt to improve the goods and services produced per unit of salt discharged to freshwater resources (Bhide et al. 2021).

### Diagnosing FSS impacts along flowpaths in energy infrastructure

FSS impacts on infrastructure mount over time with economic consequences. There may be many externalized costs that accrue due to salt damage such as long-term rusting, corrosion, and loss of ecosystem services caused by road salts and other contributors to FSS (sensu Edwards 2004). Thermoelectric power plants need freshwater from rivers to aid in the process of producing reliable energy (Badr et al. 2012), with salts leading to increased formation of scale (Ca and Mg) in pipes during the evaporative cycles associated with steam generation and cooling (Kavitha et al. 2011). The economic consequences of corrosion, pitting, and scaling of infrastructure across varying FSS stages, climate, and other state factors warrant research.

### Diagnosing FSS impacts along stormwater management infrastructure flowpaths

In addition, billions of dollars are spent on stormwater management infrastructure and stream and river restoration. Restoration and stormwater management features may also be susceptible to unintended consequences of FSS in directing deicer salts to groundwater, but also contaminant retention, release, and mobilization (Fig. 4) (Kaushal et al. 2022). Design and implementation practices require further study, particularly in regard to selecting the most appropriate soil and sediment minerals to promote ion retention and mitigate potential negative impacts on stormwater infrastructure (Kaushal et al. 2022).

### Modeling diverse flowpaths through catchments and legacy pollution sources

Once released, salt ions move through a watershed along diverse flowpaths with a broad range of transit times (i.e., the time it takes the water and associated salt ion to travel from its point of origin in the watershed to a stream or other receiving water of interest) (Hrachowitz et al. 2016). This diversity of flowpaths and transit times implies that actions taken today to reduce salt loads may take years or decades to manifest as lower ion concentrations in downstream receiving waters (Ilampooranan et al. 2019). A parsimonious framework for modeling this process represents ion mass loading out of a watershed m(t) (units of mass per time) as a convolution of the rate ions enter the watershed over time $J_{\text{s}}(t)$ (units of ion mass per time) and a probability density function f(τ) (units of inverse time) for the fraction of water flowing out of the watershed with transit times close to τ (units of time) (Kirchner et al. 2000):

$$m(t)=\int_{0}^{t}J_{s}(t-\tau )f(\tau )d\tau$$

Conceptually, this equation implies that the flux of ions out of a watershed depends on all human activities, up to the present moment, that have historically deposited ions on the landscape filtered through the set of hydrological processes (rainfall patterns, vadose zone hydrology, hydrogeological setting, and so on) that control ion movement through the catchment (Hrachowitz et al. 2016). Van Meter et al. (2017, 2018) adopted a modified version of this convolution framework to evaluate legacy nitrogen pollution in the Mississippi River basin, focusing specifically on the potential tradeoff between the cost of nutrient management interventions and time lags associated with meeting load reduction targets in the Gulf of Mexico (as represented by Pareto fronts, *see* fig. 3B in Van Meter et al. 2018). A similar approach could be adopted to assess the likely costs, benefits, and time lags associated with various salt management strategies, although this will require developing credible approaches for estimating the source and residence time distribution functions found in Eq. 1. The former requires understanding the timing, magnitude, and spatial distribution of salt inputs from nonpoint sources, such as road deicers, along with characterizing ion exchange and other reactions that act to retain or retard salts as they move through biogeochemically active soils. The latter requires adopting a mathematical form for the transit time distribution (which in turn reflects certain assumptions about the underlying physics of ion transport through the watershed; Botter et al. 2011; Rinaldo et al. 2011; Benettin et al. 2013; Kim et al. 2016; Lutz et al. 2018), as well as accounting for nonstationarity arising from short- and long-term changes in many of the state factors already discussed, including precipitation and evapotranspiration, time-varying groundwater storage, human activities, and the cumulative impact these processes have on surface and subsurface transport pathways (Pangle et al. 2017; Rodriguez et al. 2018). Many fundamental insights into catchment hydrology have come from studying how conservative ions, such as chloride, move through pristine catchments from rainfall to streams (Kirchner et al. 2000; Harman 2015). Extending these insights to urban and agriculturally impacted watersheds has the potential to improve process-level knowledge about ion pollution in human-disturbed landscapes and provide practical guidance to managers tasked with reversing inland freshwater salinization.

### Sensors along flowpaths can identify FSS stages from diagnosis to prognosis to cure

A research frontier is to use sensor data measuring conductivity to diagnose FSS stages by developing relationships between conductivity and salt ions and chemical cocktails along flowpaths. Sensor proxies can directly quantify the magnitude, frequency, and duration of exceedances in salinity thresholds for critical ecosystem services (e.g., protecting drinking water, aquatic life, infrastructure, and food production) (Fig. 5). Conductivity is a robust proxy for a wide variety of chemical constituents such as Cl^−^, Na^+^, NO_3_^−^, and certain metals (e.g., Sr, Mn, Cu) (Haq et al. 2018; Moore et al. 2020; Morel et al. 2020; Galella et al. 2021; Kaushal et al. 2021). A critical need for diagnosing and regulating FSS is the development of probabilistic models based on sensor data and long-term monitoring data to predict FSS impacts on drinking water, aquatic life, infrastructure, food production, etc.

## Conclusions

Anticipating and predicting the future of FSS requires a better understanding of diverse sources of chemical cocktails, proposed state factors and evolving stages of biological, geological, and chemical interactions. Understanding the individual roles and interactions among the five proposed state factors, and how they contribute to the development and spread of FSS, can help clarify the complexity and diversity of salinization effects and the etiology of environmental, infrastructure, and aquatic and human health consequences. For example, droughts and sea level rise are dependent on climate as a state factor, and clearly contribute to progression of FSS. Conceptual, statistical, and mechanistic models are needed to develop smart monitoring programs, improve diagnosis, and explore the causes and consequences of FSS, and identify the most promising mitigation strategies, all while taking into account past, present, and future trends. Analyzing interactions among state factors and stages of FSS across regions can indicate freshwaters, habitats, and infrastructure at risk and help identify and prioritize proper local management strategies.

## Supplementary Material

Supplement1

## Figures and Tables

**Table 1.** Potential impacts of increasing base cation and anions from FSS on ecosystem functions and processes. The table is grouped based on nutrient cycles or elements (light blue header). Mechanisms/processes illustrate the interactions between the ion from FSS and other elemental systems. Ecosystem function and processes explains impact of interacting ion process on the ecosystem. Charges are given for the base cations and anions. Charges are not indicated for the trace metals with various oxidation states. References related to elemental interactions and impacts are listed in Supporting Information.

Increased base cation/anion from FSS	Potential interactions with other elements	Mechanisms/processes of ion interactions	Impacts on ecosystem functions and processes	References
Trace element interactions				
Ca^2+^, Mg^2+^	Cd, Zn, Cu	Ca^2+^ and Mg^2+^ competes for binding and exchange sites on aquatic organisms with trace metals	Decreases toxicity of metals in organisms	[1, 2]
Ca^2+^, Mg^2+^	Fe	Rapid clay flocculation with increase saline water to remove dissolved and colloidal trace metals from the water column	Increases water clarity, increases photosynthesis in surface waters	[3, 4]
Cl^−^	Hg	Ion exchange of NaCl from lake stratification releases Hg	Hg bioaccumulation in aquatic food web	[5, 6, 7]
Cl^−^, SO_4_^2−^	Cu, Cu, Pb, Pb, Fe	Galvanized steel/iron pipes are susceptible to corrosion, leaches Pb and Fe. Cu pipes susceptible to pitting corrosion	Toxicity to freshwater organisms, growth inhibitor	[8, 9, 10]
Na^+^, K^+^, Ca^2+^, Mg^2+^	Mn, Sr, Cu, Hg, Zn	Displace and mobilize from exchange sites on colloids	Bioaccumulation of Zn, Cu, Cd, Cr, Pb, Ni in viscera of fish	[11, 12, 13]
Na^+^, Cl^−^	Ra	Cation exchange between Na^+^ and Ra; solubility of Ra increases from formation of RaCl^+^ complexes in saline water	Ra bioaccumulation in some aquatic organisms	[14, 15, 16]
Cl^−^, NO_3_^−^	U	Competition of the carbonate-uranyl-ion with anions (i.e., Cl^−^, NO_3_^−^, CO_3_^2−^) increase solubility of U	U accumulation in leaf litter; impairs physiological functions in aquatic organisms	[16, 17, 18]
Nitrogen cycle interactions				
Na^+^	N, P, NH_4_^+^	Reduction of NH_4_^+^ by Na^+^ cause ion exchange and flushing of NH_4_^+^-N	Stimulates eutrophication	[2, 19, 20, 21]
Na^+^	N, NH_4_^+^, NO_3_^−^	Increase the buffering capacity of soil system	Changes pH; enhanced nitrification of influxes of NH_4_^+^-N to soils	[19, 22, 23, 24]
Na^+^, K^+^, Ca^2+^, Mg^2+^	NO_3_^−^	Mobilizes labile protein-like fluorophores to stream from sediments	Stimulates NO_3_^−^ biological uptake; anoxic conditions for N removal during decomposition	[20, 25]
Na^+^	NH_4_^+^	Increase Na flushes NH4+ from cation exchange sites and increases NH4+ mobility from competitive ion-exchange effects from weak neutral soluble salts	Shortages of available N downstream; creates an NH_4_^+^ limitation which generates plant stress and growth suppression	[19, 26]
Na^+^, Cl^−^	NO_3_^−^, NH_4_^+^	Disrupts mineral inorganic fraction of NO_3_^−^-N and NH_4_^+^-N which increased soil pH	Long-term salt exposure-controlled rates of microbial N transformation processes	[19, 27]
Organic matter interactions				
Na+	Ca^2+^, Mg^2+^, organic matter	Na^+^ replaces Ca^2+^ and Mg^2+^ and destroys cation bridging; accelerated leaching of Ca^2+^ and Mg^2+^ from soil exchange sites due to enhanced competition with Na^+^	Destroys soil aggregate by reducing bonding with organic matter and leads to erosion	[24, 28, 29, 30]
Cl^−^	Organic matter	Decreased b and a chlorophyll and reduce CO_2_ uptake and photosynthetic capacity	Reduces photosynthetic activity; suppresses plant growth	[29, 31]
Ca^2+^, Mg^2+^	Organic matter	Aggregation and flocculation of suspended matter	Removes particles from the water column, increases light penetration and photosynthesis, and causes cyanobacteria blooms	[3, 4, 32]
Na^+^, Cl^−^, NaHCO_3_	Organic matter	Controversial—salinity driven selection of less efficient bacteria decomposers; could also be association with nutrient availability, litter type, temperature	Lowers the efficiency to breakdown leaf litter	[33, 34]
SO_4_^2−^	Organic matter	Enhances organic matter breakdown via C mineralization	Increases CO_2_ emissions in wetlands	[35, 36]
Na^+^, Cl^−^	Organic matter	Displacement of Ca^2+^ and Mg^2+^ ions from cation exchange sites by Na^+^ ions	Long-term soil pH increase; enhances solubility and mineralization of organic N	[19, 37]
Na^+^, Cl^−^	Organic matter	Mobile anion effect which reduces organic matter solubility	Short-term pH suppression	[19]
Na^+^, Cl^−^	Organic matter	Short- and long-term Na^+^ induced dispersion	Causes organic matter to leach from soils and into drainage waters	[19, 37]
Carbon cycle intera				
Na^+^, Cl^−^	ctions C	High salinity levels cause energy demand for ion transportation to maintain homeostasis in cells	Harmful algae blooms	[2, 38, 39]
Na^+^, Cl^−^	Dissolved organic carbon (DOC)	Mobilization of DOC due to the sodium dispersion/ “salting-in” effect	Stimulates microbial growth and activity; reduces dissolved organic nitrogen (e.g., NO_3_^−^)	[20, 40]
K^+^	C	Increase K^+^ provides a limiting nutrient in plant tissue	Increases terrestrial primary productivity	[41, 42]
HCO3^−^	C	More C in water so increases C taken up by phytoplankton and algae	Increases aquatic primary productivity	[42, 43, 44]
Na^+^, Cl^−^	Dissolved inorganic carbon (DIC)	Mobilization of DIC with organic carbon mineralization	Increase in alkalinity	[20, 45]
Phosphorus cycle interactions				
Na^+^, Mg^2+^, Cl^−^	PO_4_^3−^	Release of P associated with oxide surfaces or dissolution of Ca-P phases	Accelerates plant growth, algal blooms, eutrophication	[2, 20, 46, 47]
SO_4_^2−^, Cl^−^	PO_4_^2−^	SO_4_^2−^ replaces PO_4_^3−^ due to the higher ionic strength; more P released from addition of SO_4_^2−^ than Cl^−^ due to charge	Eutrophication	[2, 46]
Na^+^, Mg^2+^, K^+^, SO_4_^2−^	P	Releases P from oxyhydroxides in sediment and soils	Eutrophication	[10, 42, 45]
Na^+^, Cl^−^	Fe(Al-) phosphate complexes	Decreases stability of the colloidal humic Fe(Al-) phosphate complexes	Reduced releases of soluble reactive phosphorus from sediment and soils	[20, 48]
Na^+^, Cl^−^	P	Increased SO_4_^2−^ concentration which is an alternative terminal electron acceptor in anaerobic sediments, increased P mineralization in wetlands	Internal eutrophication	[36, 49]
Sulfur cycle interacti				
Na^+^, Cl^−^	ons S	Sulfate-reducing bacteria use SO_4_^2−^ for anaerobic respiration in saline groundwater	Elevated levels of sulfate, dissolved iron, dissolved nitrate	[4, 50]
SO_4_^2−^	HS^−^	Stimulate microbial sulphate reduction	Phytoxic, inhibits nitrification	[1, 51]
SO_4_^2−^	HS^−^	Interferes with Fe-P bonds and mobilizes P	Leads to eutrophication	[1, 3, 49]
Hydrogen cycle interactions				
Na^+^	H^+^	Mobilizes H^+^ via ion exchange	Temporary episodic acidification or long-term alkalinization	[42, 45]
Cl^−^	H^+^	Release H^+^ due to increased ionic strength caused by Cl^−^	Initial decrease in pH of the soil; impact edge charge on clays and can cause organic matter-cation colloid dispersion	[50, 52]
Na^+^, K^+^, Ca^2+^, Mg^2+^	H^+^	Increase concentrations of dissolved salts and displacement of base cations on soil exchange sites raises pH	Increases DOC, NH_3_ toxicity, stimulate NO_3_^−^ production	[2, 53, 54, 55]
Oxygen cycle interactions				
Na^+^, Cl^−^	O_2_	Layers of different densities cause lake stratification and meromictic lakes	Diminishes habitat of the deeper stratified waters due to anoxia; impact spring plankton dynamics and alters primary production	[4, 7, 56]
Na^+^, Cl^−^	O_2_	Solubility of dissolved oxygen (DO) decreases with increasing salinity	Hypoxic (DO < 3 mg L^−1^) or anoxic conditions	[57, 58, 59]
Na^+^, Cl^−^	O_2_	Low oxygen conditions allow release of phosphorus due to anaerobic bacteria in sediments	If/when lake mixes, can cause harmful cyanobacterial blooms	[26, 60, 61]

## Data Availability

Data and metadata are available at https://doi.org/10.5061/dryad.kd51c5b77.

## Scientific Significance Statement

Freshwater salinization syndrome (FSS) refers to a group of interactive physical, chemical, and biological impacts caused by increased ion concentrations, which break down natural, built, and social systems. Less is known regarding fundamental factors controlling FSS, particularly due to complex interactions among multiple ions and contaminants. There may be progressive stages of severity influenced by transitions from episodic pulses of ions to chronic long-term salinization and shifts in ecosystem processes, services, and states. We synthesize global drivers of FSS (state factors) and analyze data on stream salinization pulses and trends and rising river temperature trends. We hypothesize five fundamental state factors that contribute to stages of FSS and develop a framework for predicting, monitoring, and restoring freshwaters at risk. Our state factors and stages approach collapses the expanding diversity of FSS drivers and chemical contaminants into understandable categories to advance future research on diagnosing interactive impacts and predicting ecosystem recovery.
