# Supplementary material for: Five state factors control progressive stages of freshwater salinization syndrome
Source: Limnol Oceanogr Lett. Author manuscript; Available in PMC 2024 Feb 1. (PMC10395323; doi:10.1002/lol2.10248)
Supplement: Supplement1 [file NIHMS1876081-supplement-Supplement1.docx]

**Supporting Information**

**Figure 1. Rising Temperatures of Global Freshwaters**

| **Complete Reference** | **Latitude** | **Longitude** | **River/Water Body** | **Period of Record** | **Data/Sampling Frequency** | **Digitized Figure Data Originates From** |
| --- | --- | --- | --- | --- | --- | --- |
| Casola, J.H., J.E. Kay, A.K. Snover, R.A. Norheim, L. C. Whitely Binder and the Climate Impacts Group. 2005. Climate Impacts on Washington’s Hydropower, Water Supply, Forests, Fish, and Agriculture. A report prepared for King County (Washington) by the Climate Impacts Group (Center for Science in the Earth System, Joint Institute for the Study of the Atmosphere and Ocean, University of Washington, Seattle). | 49.1 | -123.167 | Fraser River, Canada | 1952-1996 | Mean Annual Summer Temperature | Figure 10 |
| Kocan, R., P. Hershberger and J. Winton. 2003. Effects of Ichthyophonus on Survival and Reproductive Success of Yukon River Chinook Salmon. Federal Subsistence Fisheries Resource Monitoring Program, Final Project Report No. FIS 01-200. U. S. Fish and Wildlife Service, Office of Subsistence Management, Fisheries Information Services Division, Anchorage, Alaska. | 62.533 | -163.9 | Yukon River, Canada | 1975-2002 | Mean Annual June Temperature | Figure 16 |
| Johnson, Stephanie L., and Heinz G. Stefan. “Indicators of Climate Warming in Minnesota: Lake ICE Covers and Snowmelt Runoff.” Climatic Change, vol. 75, no. 4, 2006, pp. 421–453., doi:10.1007/s10584-006-0356-0. | 35.514706 | -89.912506 | Mississippi River, United States | 1975-2002 | Annual Average Temperature | Figure 8 |
| Kaushal, Sujay S, et al. “Rising Stream and River Temperatures in the United States.” Frontiers in Ecology and the Environment, vol. 8, no. 9, 2010, pp. 461–466., doi:10.1890/090037. | 43.9419 | -71.7257 | Hubbard Brook, United States | 1965-2005 | Annual Average Temperature | Figure 1 |
| Kaushal, Sujay S, et al. “Rising Stream and River Temperatures in the United States.” Frontiers in Ecology and the Environment, vol. 8, no. 9, 2010, pp. 461–466., doi:10.1890/090037. | 42.080068 | -73.930390 | Hudson River, United States | 1905-1993 | Annual Average Temperature | Figure 1 |
| Kaushal, Sujay S, et al. “Rising Stream and River Temperatures in the United States.” Frontiers in Ecology and the Environment, vol. 8, no. 9, 2010, pp. 461–466., doi:10.1890/090037. | 40.29245 | -74.86848 | Delaware River, United States | 1965-2005 | Annual Average Temperature | Figure 1 |
| Kaushal, Sujay S, et al. “Rising Stream and River Temperatures in the United States.” Frontiers in Ecology and the Environment, vol. 8, no. 9, 2010, pp. 461–466., doi:10.1890/090037. | 39.82572 | -75.5732 | Brandywine Creek, United States | 1972-2006 | Annual Average Temperature | Figure 1 |
| Kaushal, Sujay S, et al. “Rising Stream and River Temperatures in the United States.” Frontiers in Ecology and the Environment, vol. 8, no. 9, 2010, pp. 461–466., doi:10.1890/090037. | 38.439373 | -77.302759 | Potomac River, United States | 1920-1999 | Annual Average Temperature | Figure 1 |
| Kaushal, Sujay S, et al. “Rising Stream and River Temperatures in the United States.” Frontiers in Ecology and the Environment, vol. 8, no. 9, 2010, pp. 461–466., doi:10.1890/090037. | 47.44304387279977 | -114.33724411548383 | Flathead River, United States | 1979-2005 | Annual Average Temperature | Figure 1 |
| Kaushal, Sujay S, et al. “Rising Stream and River Temperatures in the United States.” Frontiers in Ecology and the Environment, vol. 8, no. 9, 2010, pp. 461–466., doi:10.1890/090037. | 42.65046376265154 | -123.58786456729207 | Rogue River, Oregon, United States | 1979-2005 | Annual Average Temperature | Figure 1 |
| Isaak, D. J., et al. “Climate Change Effects on Stream and River Temperatures across the Northwest U.S. from 1980–2009 and Implications for Salmonid Fishes.” Climatic Change, vol. 113, no. 2, 2011, pp. 499–524., doi:10.1007/s10584-011-0326-z. | 48.15776 | -115.78408 | South Fork Bull Run River, Orgeon | 1980-2009 | Raw Seasonal Temperature (Winter): Temperatures offset by +4 Deg C on plot* | Figure 2 |
| Bartholow, J. M. “Recent Water Temperature Trends in the Lower Klamath River, California.” North American Journal of Fisheries Management, vol. 25, no.1, 2005, pp. 152–162., doi:[10.1577/M04-007.1](https://doi.org/10.1577/M04-007.1) | 42 | -123.03 | Klamath River | 1962-2001 (Water Years) | Mean Monthly Water Year Temperature | Figure 2 |
| Vollmer, M. K. et al. “Deep-water warming trend in Lake Malawi, East Africa.” Limnology and Oceanography, vol. 50, 2005, pp. 727–732. | -12 | 34.5 | Lake Malawi | 1939 -2000 | Mean Annual Temperature (100m depth) | Figure 2 |
| Webb, B. W. “Trends in stream and river temperature.” Hydrological Processes, vol. 10 no.2, 1996, pp. 205–226. [https://doi.org/10.1002/(SICI)1099-1085(199602)10:2<205::AID-HYP358>3.0.CO;2-1](https://doi.org/10.1002/(SICI)1099-1085(199602)10:2%3c205::AID-HYP358%3e3.0.CO;2-1) | 48.04 | 14.129719 | Kremsmünster River | 1901-1990 | Mean Annual Water Temperature | Figure 2 |
| Webb, B. W., & Nobilis, F. “Long-term changes in river temperature and the influence of climatic and hydrological factors.” Hydrological Sciences Journal, vol. 52, no.1, 2007, pp. 74-85. | 47.2794 | 12.4793 | Salzach River (Headwaters Site) | 1901-2000 | Mean Annual Water Temperature | Figure 2A |
| Ye, F. & Kameyama, S. “Long-term nationwide spatiotemporal changes of freshwater temperature in Japan during 1982–2016.” Journal of Environmental Management vol.281, 2021. | 34.648442 | 137.7935 | Tenryu River | 1982-2016 | Mean Annual Water Temperature | Figure 4 |

**Figure 2: State Factors Forming Freshwater Salinization Syndrome**

**These references correspond to superscripts from Figure 2 demonstrating the state factors and causes of Freshwater Salinization Syndrome, such as climate, geology, human activities, flowpaths, and time. These are examples from the literature which do not address FSS as a concept, but describe the increase of one/more salt ions and the impacts. The number in front of the reference below corresponds the number in the figure on the left-hand side of each example. The references are listed numerically below.**

[1] Kaushal et al. 2018b [2] Guo et al. 2015 [3] Cañedo-Argüelles 2020 [4] Nield et al. 2008 [5] Liu et al. 2013 [6] Sereda et al. 2011 [7] Kaushal et al. 2019 [8] Kaushal et al. 2021 [9] Kaushal et al. 2014 [10] Zhang et al. 2009 [11] Wang et al. 2019 [12] Jobbágy and Jackson 2004 [13] Shanley 1994 [14] Ostendorf et al. 2009 [15] Walter et al. 2017 [16] Vengosh 2005 [17] Kaushal et al. 2017 [18] Kaushal et al. 2005 [19]Kelly et al. 2008 [20] Kaushal et al. 2018a [21] Raymond et al. 2008 [22] Bhide et al. 2021 [23] Tjandraatmadja et al. 2010 [24] Kaushal et al. 2021 [25] Meybeck and Helmer 1989 [26] Rengasamy 2006 [27] Bouzourra et al. 2015 [28] Stets et al. 2018 [29] Kaushal et al. 2017 [30] Kaushal and Belt 2012 [31] Micklin and Aladin 2008 [32] Paul et al. 2019 [33] Schulz and Cañedo-Argüelles 2019 [34] Tully et al. 2019 [35] Galella et al. 2021) [36] Hintz et al. 2017 [37] Langan et al. 1995 [38] Ouimet and Duchesne 2005 [39] David et al. 2016 [40] Haq et al. 2018 [41] Micklin 2016

**References for Figure 2**

Bhide, S. V., S. B. Grant, E. A. Parker, and others. 2021. Addressing the contribution of indirect potable reuse to inland freshwater salinization. Nat. Sustain. doi:10.1038/s41893-021-00713-7

Bouzourra, H., R. Bouhlila, L. Elango, F. Slama, and N. Ouslati. 2015. Characterization of mechanisms and processes of groundwater salinization in irrigated coastal area using statistics, GIS, and hydrogeochemical investigations. Environ. Sci. Pollut. Res. **22**: 2643–2660. doi:10.1007/s11356-014-3428-0

Cañedo-Argüelles, M. 2020. A review of recent advances and future challenges in freshwater salinization.doi:10.23818/limn.39.13

David, M. B., C. A. Mitchell, L. E. Gentry, and R. K. Salemme. 2016. Chloride Sources and Losses in Two Tile-Drained Agricultural Watersheds. J. Environ. Qual. **45**: 341–348. doi:https://doi.org/10.2134/jeq2015.06.0302

Galella, J. G., S. S. Kaushal, K. L. Wood, J. E. Reimer, and P. M. Mayer. 2021. Sensors track mobilization of `chemical cocktails’ in streams impacted by road salts in the Chesapeake Bay watershed. Environ. Res. Lett. **16**: 035017. doi:10.1088/1748-9326/abe48f

Guo, M., X. Zhou, J. Li, W. Wu, and Y. Chen. 2015. Assessment of the salinization processes in the largest inland freshwater lake of China. Stoch. Environ. Res. Risk Assess. **29**: 1823–1833. doi:10.1007/s00477-014-0995-z

Haq, S., S. S. Kaushal, and S. Duan. 2018. Episodic salinization and freshwater salinization syndrome mobilize base cations, carbon, and nutrients to streams across urban regions. Biogeochemistry **141**: 463–486.

Hintz, W. D., B. M. Mattes, M. S. Schuler, D. K. Jones, A. B. Stoler, L. Lind, and R. A. Relyea. 2017. Salinization triggers a trophic cascade in experimental freshwater communities with varying food-chain length. Ecol. Appl. **27**: 833–844. doi:https://doi.org/10.1002/eap.1487

Jobbágy, E. G., and R. B. Jackson. 2004. Groundwater use and salinization with grassland afforestation. Glob. Change Biol. **10**: 1299–1312. doi:https://doi.org/10.1111/j.1365-2486.2004.00806.x

Kaushal, S. S., and K. T. Belt. 2012. The urban watershed continuum: evolving spatial and temporal dimensions. Urban Ecosyst. **15**: 409–435. doi:10.1007/s11252-012-0226-7

Kaushal, S. S., S. Duan, T. R. Doody, and others. 2017. Human-accelerated weathering increases salinization, major ions, and alkalinization in fresh water across land use. Appl. Geochem. **83**: 121–135. doi:10.1016/j.apgeochem.2017.02.006

Kaushal, S. S., A. J. Gold, S. Bernal, and others. 2018a. Watershed ‘chemical cocktails’: forming novel elemental combinations in Anthropocene fresh waters. Biogeochemistry **141**: 281–305. doi:10.1007/s10533-018-0502-6

Kaushal, S. S., P. M. Groffman, G. E. Likens, K. T. Belt, W. P. Stack, V. R. Kelly, L. E. Band, and G. T. Fisher. 2005. Increased salinization of fresh water in the northeastern United States. Proc. Natl. Acad. Sci. **102**: 13517–13520.

Kaushal, S. S., G. E. Likens, M. L. Pace, and others. 2019. Novel ‘chemical cocktails’ in inland waters are a consequence of the freshwater salinization syndrome. Philos. Trans. R. Soc. B Biol. Sci. **374**: 20180017. doi:10.1098/rstb.2018.0017

Kaushal, S. S., G. E. Likens, M. L. Pace, and others. 2021. Freshwater salinization syndrome: from emerging global problem to managing risks. Biogeochemistry. doi:10.1007/s10533-021-00784-w

Kaushal, S. S., G. E. Likens, M. L. Pace, R. M. Utz, S. Haq, J. Gorman, and M. Grese. 2018b. Freshwater salinization syndrome on a continental scale. Proc. Natl. Acad. Sci. **115**: E574–E583. doi:10.1073/pnas.1711234115

Kaushal, S. S., W. H. McDowell, and W. M. Wollheim. 2014. Tracking evolution of urban biogeochemical cycles: past, present, and future. Biogeochemistry **121**: 1–21. doi:10.1007/s10533-014-0014-y

Kelly, V. R., G. M. Lovett, K. C. Weathers, S. E. G. Findlay, D. L. Strayer, D. J. Burns, and G. E. Likens. 2008. Long-Term Sodium Chloride Retention in a Rural Watershed: Legacy Effects of Road Salt on Streamwater Concentration. Environ. Sci. Technol. **42**: 410–415. doi:10.1021/es071391l

Langan, S. J., H. U. Sverdrup, and M. Coull. 1995. The calculation of base cation release from the chemical weathering of Scottish soils using the profile model. **4**: 6.

Liu, H., Y. Yin, S. Piao, F. Zhao, M. Engels, and P. Ciais. 2013. Disappearing Lakes in Semiarid Northern China: Drivers and Environmental Impact. Environ. Sci. Technol. **47**: 12107–12114. doi:10.1021/es305298q

Meybeck, M., and R. Helmer. 1989. The quality of rivers: From pristine stage to global pollution. Glob. Planet. Change **1**: 283–309. doi:10.1016/0921-8181(89)90007-6

Micklin, P. 2016. The future Aral Sea: hope and despair. Environ. Earth Sci. **75**: 844. doi:10.1007/s12665-016-5614-5

Micklin, P., and N. V. Aladin. 2008. Reclaiming the Aral Sea. Sci. Am. **298**: 64–71.

Nield, D. A., C. T. Simmons, A. V. Kuznetsov, and J. D. Ward. 2008. On the evolution of salt lakes: Episodic convection beneath an evaporating salt lake. Water Resour. Res. **44**. doi:https://doi.org/10.1029/2007WR006161

Ostendorf, D. W., B. Xing, and N. Kallergis. 2009. Cation exchange in a glacial till drumlin at a road salt storage facility. J. Contam. Hydrol. **106**: 118–130. doi:10.1016/j.jconhyd.2009.02.002

Ouimet, R., and L. Duchesne. 2005. Base cation mineral weathering and total release rates from soils in three calibrated forest watersheds on the Canadian Boreal Shield. Can. J. Soil Sci. **85**: 245–260. doi:10.4141/S04-061

Paul, M. J., R. Coffey, J. Stamp, and T. Johnson. 2019. A Review of Water Quality Responses to Air Temperature and Precipitation Changes 1: Flow, Water Temperature, Saltwater Intrusion. JAWRA J. Am. Water Resour. Assoc. **55**: 824–843. doi:https://doi.org/10.1111/1752-1688.12710

Raymond, P. A., N.-H. Oh, R. E. Turner, and W. Broussard. 2008. Anthropogenically enhanced fluxes of water and carbon from the Mississippi River. Nature **451**: 449–452.

Rengasamy, P. 2006. World salinization with emphasis on Australia. J. Exp. Bot. **57**: 1017–1023. doi:10.1093/jxb/erj108

Schulz, C.-J., and M. Cañedo-Argüelles. 2019. Lost in translation: the German literature on freshwater salinization. Philos. Trans. R. Soc. B Biol. Sci. **374**: 20180007. doi:10.1098/rstb.2018.0007

Sereda, J., M. Bogard, J. Hudson, D. Helps, and T. Dessouki. 2011. Climate warming and the onset of salinization: Rapid changes in the limnology of two northern plains lakes. Limnologica **41**: 1–9. doi:10.1016/j.limno.2010.03.002

Shanley, J. B. 1994. Effects of Ion Exchange on Stream Solute Fluxes in a Basin Receiving Highway Deicing Salts. J. Environ. Qual. **23**: 977–986. doi:https://doi.org/10.2134/jeq1994.00472425002300050019x

Stets, E. G., C. J. Lee, D. A. Lytle, and M. R. Schock. 2018. Increasing chloride in rivers of the conterminous U.S. and linkages to potential corrosivity and lead action level exceedances in drinking water. Sci. Total Environ. **613–614**: 1498–1509. doi:10.1016/j.scitotenv.2017.07.119

Tjandraatmadja, G., C. Pollard, C. Sheedy, and Y. Gozukra. 2010. Sources of contaminants in domestic wastewater: nutrients and additional elements from household products. CSIRO: water for a healthy country national research flagship.

Tully, K., K. Gedan, R. Epanchin-Niell, and others. 2019. The Invisible Flood: The Chemistry, Ecology, and Social Implications of Coastal Saltwater Intrusion. BioScience **69**: 368–378. doi:10.1093/biosci/biz027

Vengosh, A. 2005. Salinization and Saline, p. 333. *In* Environmental geochemistry.

Walter, J., R. Chesnaux, V. Cloutier, and D. Gaboury. 2017. The influence of water/rock − water/clay interactions and mixing in the salinization processes of groundwater. J. Hydrol. Reg. Stud. **13**: 168–188. doi:10.1016/j.ejrh.2017.07.004

Wang, H.-Y., H.-M. Guo, W. Xiu, J. Bauer, G.-X. Sun, X.-H. Tang, and S. Norra. 2019. Indications that weathering of evaporite minerals affects groundwater salinity and As mobilization in aquifers of the northwestern Hetao Basin, China. Appl. Geochem. **109**: 104416. doi:10.1016/j.apgeochem.2019.104416

Zhang, S., X. X. Lu, H. Sun, J. Han, and D. L. Higgitt. 2009. Major ion chemistry and dissolved inorganic carbon cycling in a human-disturbed mountainous river (the Luodingjiang River) of the Zhujiang (Pearl River), China. Sci. Total Environ. **407**: 2796–2807. doi:10.1016/j.scitotenv.2008.12.036

**Figure 3 Connecting Common FSS Causes to Consequences**

**These references correspond to superscripts in Figure 3 in the main text linking the causes and consequences of Freshwater Salinization Syndrome based on the state factors. These are examples from the literature which do not address FSS as a concept, but describe the increase of one/more salt ions and the impacts. The number in front of the reference is the number in the figure on the left-hand side of each example. These are listed numerically below.**

[1] Kaushal et al. 2017 [2] Kaushal et al. 2019 [3] Kaushal et al. 2018a [4] Galella et al. 2021 [5] Bernhardt and Palmer 2011 [6] Hopkins et al. 2013 [7] Haq et al. 2018 [8] Kaushal et al. 2021 [9] Stets et al. 2018a [10] Kaushal et al. 2020 [11] Cañedo-Argüelles 2020 [12] Schuler et al. 2019 [13] Vengosh 2005 [14] Jeppesen et al. 2015 [15] Simpson et al. 2010 [16] Simpson et al. 2010 [17] Vithana et al. 2021 [18] Novotny and Stefan 2012 [19] Stetler et al. 2021 [20] Stets et al. 2018b [21] Lazur et al. 2020 [22] Acosta et al. 2011 [23] Wilts et al. 2018 [24] Shammas and Wang 2015 [25] Wiest et al. 2011 [26] Letterman and AWW Association 1999 [27] Tzoupanos and Zouboulis 2008 [28] Semerjian and Ayoub 2003 [29] Bhide et al. 2021 [30] Overbo et al. 2021 [31] Gutierrez et al. 2010

**References for Figure 3**

Acosta, J. A., B. Jansen, K. Kalbitz, A. Faz, and S. Martínez-Martínez. 2011. Salinity increases mobility of heavy metals in soils. Chemosphere **85**: 1318–1324. doi:10.1016/j.chemosphere.2011.07.046

Bernhardt, E. S., and M. A. Palmer. 2011. The environmental costs of mountaintop mining valley fill operations for aquatic ecosystems of the Central Appalachians: Mountaintop mining impacts on aquatic ecosystems. Ann. N. Y. Acad. Sci. **1223**: 39–57. doi:10.1111/j.1749-6632.2011.05986.x

Bhide, S. V., S. B. Grant, E. A. Parker, and others. 2021. Addressing the contribution of indirect potable reuse to inland freshwater salinization. Nat. Sustain. doi:10.1038/s41893-021-00713-7

Bouzourra, H., R. Bouhlila, L. Elango, F. Slama, and N. Ouslati. 2015. Characterization of mechanisms and processes of groundwater salinization in irrigated coastal area using statistics, GIS, and hydrogeochemical investigations. Environ. Sci. Pollut. Res. **22**: 2643–2660. doi:10.1007/s11356-014-3428-0

Cañedo-Argüelles, M. 2020. A review of recent advances and future challenges in freshwater salinization.doi:10.23818/limn.39.13

David, M. B., C. A. Mitchell, L. E. Gentry, and R. K. Salemme. 2016. Chloride Sources and Losses in Two Tile-Drained Agricultural Watersheds. J. Environ. Qual. **45**: 341–348. doi:https://doi.org/10.2134/jeq2015.06.0302

Galella, J. G., S. S. Kaushal, K. L. Wood, J. E. Reimer, and P. M. Mayer. 2021. Sensors track mobilization of `chemical cocktails’ in streams impacted by road salts in the Chesapeake Bay watershed. Environ. Res. Lett. **16**: 035017. doi:10.1088/1748-9326/abe48f

Guo, M., X. Zhou, J. Li, W. Wu, and Y. Chen. 2015. Assessment of the salinization processes in the largest inland freshwater lake of China. Stoch. Environ. Res. Risk Assess. **29**: 1823–1833. doi:10.1007/s00477-014-0995-z

Gutierrez, O., D. Park, K. R. Sharma, and Z. Yuan. 2010. Iron salts dosage for sulfide control in sewers induces chemical phosphorus removal during wastewater treatment. Water Res. **44**: 3467–3475. doi:10.1016/j.watres.2010.03.023

Haq, S., S. S. Kaushal, and S. Duan. 2018. Episodic salinization and freshwater salinization syndrome mobilize base cations, carbon, and nutrients to streams across urban regions. Biogeochemistry **141**: 463–486.

Hintz, W. D., B. M. Mattes, M. S. Schuler, D. K. Jones, A. B. Stoler, L. Lind, and R. A. Relyea. 2017. Salinization triggers a trophic cascade in experimental freshwater communities with varying food-chain length. Ecol. Appl. **27**: 833–844. doi:https://doi.org/10.1002/eap.1487

Hopkins, R. L., B. M. Altier, D. Haselman, A. D. Merry, and J. J. White. 2013. Exploring the legacy effects of surface coal mining on stream chemistry. Hydrobiologia **713**: 87–95. doi:10.1007/s10750-013-1494-9

Jeppesen, E., S. Brucet, L. Naselli-Flores, and others. 2015. Ecological impacts of global warming and water abstraction on lakes and reservoirs due to changes in water level and related changes in salinity. Hydrobiologia **750**: 201–227. doi:10.1007/s10750-014-2169-x

Jobbágy, E. G., and R. B. Jackson. 2004. Groundwater use and salinization with grassland afforestation. Glob. Change Biol. **10**: 1299–1312. doi:https://doi.org/10.1111/j.1365-2486.2004.00806.x

Kaushal, S. S., and K. T. Belt. 2012. The urban watershed continuum: evolving spatial and temporal dimensions. Urban Ecosyst. **15**: 409–435. doi:10.1007/s11252-012-0226-7

Kaushal, S. S., S. Duan, T. R. Doody, and others. 2017. Human-accelerated weathering increases salinization, major ions, and alkalinization in fresh water across land use. Appl. Geochem. **83**: 121–135. doi:10.1016/j.apgeochem.2017.02.006

Kaushal, S. S., A. J. Gold, S. Bernal, and others. 2018a. Watershed ‘chemical cocktails’: forming novel elemental combinations in Anthropocene fresh waters. Biogeochemistry **141**: 281–305. doi:10.1007/s10533-018-0502-6

Kaushal, S. S., P. M. Groffman, G. E. Likens, K. T. Belt, W. P. Stack, V. R. Kelly, L. E. Band, and G. T. Fisher. 2005. Increased salinization of fresh water in the northeastern United States. Proc. Natl. Acad. Sci. **102**: 13517–13520.

Kaushal, S. S., G. E. Likens, M. L. Pace, and others. 2019. Novel ‘chemical cocktails’ in inland waters are a consequence of the freshwater salinization syndrome. Philos. Trans. R. Soc. B Biol. Sci. **374**: 20180017. doi:10.1098/rstb.2018.0017

Kaushal, S. S., G. E. Likens, M. L. Pace, and others. 2021. Freshwater salinization syndrome: from emerging global problem to managing risks. Biogeochemistry. doi:10.1007/s10533-021-00784-w

Kaushal, S. S., G. E. Likens, M. L. Pace, R. M. Utz, S. Haq, J. Gorman, and M. Grese. 2018b. Freshwater salinization syndrome on a continental scale. Proc. Natl. Acad. Sci. **115**: E574–E583. doi:10.1073/pnas.1711234115

Kaushal, S. S., W. H. McDowell, and W. M. Wollheim. 2014. Tracking evolution of urban biogeochemical cycles: past, present, and future. Biogeochemistry **121**: 1–21. doi:10.1007/s10533-014-0014-y

Kaushal, S. S., K. L. Wood, J. G. Galella, and others. 2020. Making ‘chemical cocktails’ – Evolution of urban geochemical processes across the periodic table of elements. Appl. Geochem. **119**: 104632. doi:10.1016/j.apgeochem.2020.104632

Kelly, V. R., G. M. Lovett, K. C. Weathers, S. E. G. Findlay, D. L. Strayer, D. J. Burns, and G. E. Likens. 2008. Long-Term Sodium Chloride Retention in a Rural Watershed: Legacy Effects of Road Salt on Streamwater Concentration. Environ. Sci. Technol. **42**: 410–415. doi:10.1021/es071391l

Langan, S. J., H. U. Sverdrup, and M. Coull. 1995. The calculation of base cation release from the chemical weathering of Scottish soils using the profile model. **4**: 6.

Lazur, A., T. VanDerwerker, and K. Koepenick. 2020. Review of Implications of Road Salt Use on Groundwater Quality—Corrosivity and Mobilization of Heavy Metals and Radionuclides. Water. Air. Soil Pollut. **231**: 474. doi:10.1007/s11270-020-04843-0

Letterman, R. D. and AWW Association. 1999. Water quality and treatment: a handbook of community water supplies, McGraw-Hill.

Liu, H., Y. Yin, S. Piao, F. Zhao, M. Engels, and P. Ciais. 2013. Disappearing Lakes in Semiarid Northern China: Drivers and Environmental Impact. Environ. Sci. Technol. **47**: 12107–12114. doi:10.1021/es305298q

Meybeck, M., and R. Helmer. 1989. The quality of rivers: From pristine stage to global pollution. Glob. Planet. Change **1**: 283–309. doi:10.1016/0921-8181(89)90007-6

Micklin, P., and N. V. Aladin. 2008. Reclaiming the Aral Sea. Sci. Am. **298**: 64–71.

Nield, D. A., C. T. Simmons, A. V. Kuznetsov, and J. D. Ward. 2008. On the evolution of salt lakes: Episodic convection beneath an evaporating salt lake. Water Resour. Res. **44**. doi:https://doi.org/10.1029/2007WR006161

Novotny, E. V., and H. G. Stefan. 2012. Road Salt Impact on Lake Stratification and Water Quality. J. Hydraul. Eng. **138**: 1069–1080. doi:10.1061/(ASCE)HY.1943-7900.0000590

Ostendorf, D. W., B. Xing, and N. Kallergis. 2009. Cation exchange in a glacial till drumlin at a road salt storage facility. J. Contam. Hydrol. **106**: 118–130. doi:10.1016/j.jconhyd.2009.02.002

Ouimet, R., and L. Duchesne. 2005. Base cation mineral weathering and total release rates from soils in three calibrated forest watersheds on the Canadian Boreal Shield. Can. J. Soil Sci. **85**: 245–260. doi:10.4141/S04-061

Overbo, A., S. Heger, and J. Gulliver. 2021. Evaluation of chloride contributions from major point and nonpoint sources in a northern U.S. state. Sci. Total Environ. **764**: 144179. doi:10.1016/j.scitotenv.2020.144179

Paul, M. J., R. Coffey, J. Stamp, and T. Johnson. 2019. A Review of Water Quality Responses to Air Temperature and Precipitation Changes 1: Flow, Water Temperature, Saltwater Intrusion. JAWRA J. Am. Water Resour. Assoc. **55**: 824–843. doi:https://doi.org/10.1111/1752-1688.12710

Raymond, P. A., N.-H. Oh, R. E. Turner, and W. Broussard. 2008. Anthropogenically enhanced fluxes of water and carbon from the Mississippi River. Nature **451**: 449–452.

Rengasamy, P. 2006. World salinization with emphasis on Australia. J. Exp. Bot. **57**: 1017–1023. doi:10.1093/jxb/erj108

Schuler, M. S., M. Cañedo-Argüelles, W. D. Hintz, B. Dyack, S. Birk, and R. A. Relyea. 2019. Regulations are needed to protect freshwater ecosystems from salinization. Philos. Trans. R. Soc. B Biol. Sci. **374**: 20180019. doi:10.1098/rstb.2018.0019

Schulz, C.-J., and M. Cañedo-Argüelles. 2019. Lost in translation: the German literature on freshwater salinization. Philos. Trans. R. Soc. B Biol. Sci. **374**: 20180007. doi:10.1098/rstb.2018.0007

Semerjian, L., and G. M. Ayoub. 2003. High-pH–magnesium coagulation–flocculation in wastewater treatment. Adv. Environ. Res. **7**: 389–403. doi:10.1016/S1093-0191(02)00009-6

Sereda, J., M. Bogard, J. Hudson, D. Helps, and T. Dessouki. 2011. Climate warming and the onset of salinization: Rapid changes in the limnology of two northern plains lakes. Limnologica **41**: 1–9. doi:10.1016/j.limno.2010.03.002

Shammas, N. K., and L. K. Wang. 2015. Water engineering: hydraulics, distribution and treatment, John Wiley & Sons, Ltd.

Shanley, J. B. 1994. Effects of Ion Exchange on Stream Solute Fluxes in a Basin Receiving Highway Deicing Salts. J. Environ. Qual. **23**: 977–986. doi:https://doi.org/10.2134/jeq1994.00472425002300050019x

Simpson, S. L., R. W. Fitzpatrick, P. Shand, and others. 2010. Climate-driven mobilisation of acid and metals from acid sulfate soils. Mar. Freshw. Res. **61**: 129–138. doi:10.1071/MF09066

Stetler, J. T., S. Girdner, J. Mack, L. A. Winslow, T. H. Leach, and K. C. Rose. 2021. Atmospheric stilling and warming air temperatures drive long-term changes in lake stratification in a large oligotrophic lake. Limnol. Oceanogr. **66**: 954–964. doi:https://doi.org/10.1002/lno.11654

Stets, E. G., C. J. Lee, D. A. Lytle, and M. R. Schock. 2018a. Increasing chloride in rivers of the conterminous U.S. and linkages to potential corrosivity and lead action level exceedances in drinking water. Sci. Total Environ. **613–614**: 1498–1509. doi:10.1016/j.scitotenv.2017.07.119

Stets, E. G., C. J. Lee, D. A. Lytle, and M. R. Schock. 2018b. Increasing chloride in rivers of the conterminous U.S. and linkages to potential corrosivity and lead action level exceedances in drinking water. Sci. Total Environ. **613–614**: 1498–1509. doi:10.1016/j.scitotenv.2017.07.119

Tjandraatmadja, G., C. Pollard, C. Sheedy, and Y. Gozukra. 2010. Sources of contaminants in domestic wastewater: nutrients and additional elements from household products. CSIRO: water for a healthy country national research flagship.

Tully, K., K. Gedan, R. Epanchin-Niell, and others. 2019. The Invisible Flood: The Chemistry, Ecology, and Social Implications of Coastal Saltwater Intrusion. BioScience **69**: 368–378. doi:10.1093/biosci/biz027

Tzoupanos, N. D., and A. I. Zouboulis. 2008. COAGULATION-FLOCCULATION PROCESSES IN WATER/WASTEWATER TREATMENT: THE APPLICATION OF NEW GENERATION OF CHEMICAL REAGENTS. 9.

Vengosh, A. 2005. Salinization and Saline, p. 333. *In* Environmental geochemistry.

Vithana, C. L., P. A. K. Ulapane, R. Chandrajith, L. A. Sullivan, J. Bundschuh, N. Toppler, N. J. Ward, and A. Senaratne. 2021. Acid sulfate soils on the west coast of Sri Lanka: A review. Geoderma Reg. **25**: e00382. doi:10.1016/j.geodrs.2021.e00382

Walter, J., R. Chesnaux, V. Cloutier, and D. Gaboury. 2017. The influence of water/rock − water/clay interactions and mixing in the salinization processes of groundwater. J. Hydrol. Reg. Stud. **13**: 168–188. doi:10.1016/j.ejrh.2017.07.004

Wang, H.-Y., H.-M. Guo, W. Xiu, J. Bauer, G.-X. Sun, X.-H. Tang, and S. Norra. 2019. Indications that weathering of evaporite minerals affects groundwater salinity and As mobilization in aquifers of the northwestern Hetao Basin, China. Appl. Geochem. **109**: 104416. doi:10.1016/j.apgeochem.2019.104416

Wiest, M., P. Fox, W. Lee, and T. Thomure. 2011. Evaluation of Alternatives to Domestic Ion Exchange Water Softeners. Proc. Water Environ. Fed. **2011**: 4659–4671. doi:10.2175/193864711802765246

Wilts, E. M., J. Herzberger, and T. E. Long. 2018. Addressing water scarcity: cationic polyelectrolytes in water treatment and purification. Polym. Int. **67**: 799–814. doi:https://doi.org/10.1002/pi.5569

Zhang, S., X. X. Lu, H. Sun, J. Han, and D. L. Higgitt. 2009. Major ion chemistry and dissolved inorganic carbon cycling in a human-disturbed mountainous river (the Luodingjiang River) of the Zhujiang (Pearl River), China. Sci. Total Environ. **407**: 2796–2807. doi:10.1016/j.scitotenv.2008.12.036

**Table 1:**

**The number in front of each reference, listed numerically below, corresponds to numbers in the Reference column of Table 1 in the main text showing the impact of higher concentrations of base cations and ions resulting from Freshwater Salinization Syndrome on ecosystem functions and processes. The examples from the literature do not address FSS as a concept but describe increases in one or more salt ions and the impacts.**

[1] Cañedo-Argüelles et al. 2013 [2] Iglesias 2020 [3] Donnelly et al. 1997 [4] Nielsen et al. 2003 [5] Mclaughlin et al. 1996 [6] Mirlean et al. 2005 [7] Ramakrishna and Viraraghavan 2005 [8] de Oliveira-Filho et al. 2004 [9] Grosell et al. 2007 [10] Kaushal et al. 2021 [11] Liang et al. 1999 [12] Sandor et al. 2001 [13] Galella et al. 2021 [14] Bollhöfer et al. 2011 [15] McNaboe et al. 2017 [16] Lazur et al. 2020 [17] Riedel and Kübeck 2018 [18] Bergmann et al. 2018 [19] Green and Cresser 2008a [20] Duan and Kaushal 2015 [21] Kaushal et al. 2017 [22] Šimek and Cooper 2002 [23] Sahrawat 2008 [24] Kaushal et al. 2018b [25] Li et al. 2007 [26] Findlay and Kelly 2011 [27] Green et al. 2008a [28] Green and Cresser 2008b [29] Dai et al. 2012 [30] Mavi et al. 2012 [31] Nazarbeygi et al. 2011 [32] Geddes 1988 [33] Sauer et al. 2016 [34] Berger et al. 2019 [35] Marton et al. 2012 [36] van Dijk et al. 2019 [37] Green et al. 2008b [38] Jeppesen et al. 2015 [39] Lind et al. 2018 [40] Bernhardt and Likens 2002 [41] Tripler et al. 2006 [42] Kaushal et al. 2019 [43] White et al. 2013 [44] Yue et al. 2021 [45] Kaushal et al. 2018a [46] Ahmad et al. 2008 [47] Duan and Kaushal 2013 [48] Spiteri et al. 2008 [49] Smolders et al. 2006 [50] Haq et al. 2018 [51] Bernhardt and Palmer 2011 [52] Norrström and Bergstedt 2001 [53] Steele and Aitkenhead-Peterson 2011 [54] Kaushal et al. 2014 [55] Boyd et al. 2016 [56] Judd 1970 [57] Sherwood et al. 1991 [58] Best et al. 2007 [59] Hansson and Gustafsson 2011 [60] Amirbahman et al. 2003 [61] Bormans et al. 2016

**References for Table 1**

Ahmad, Z., Faridullah, H. El-Sharkawi, M. Irshad, T. Honna, S. Yamamoto, and A. S. Al-Busaidi. 2008. Changes in water-extractability of soil inorganic phosphate induced by chloride and sulfate salts. Environ Sci Pollut Res **15**: 23–26. doi:10.1065/espr2006.06.309

Amirbahman, A., A. R. Pearce, R. J. Bouchard, S. A. Norton, and J. S. Kahl. 2003. Relationship between hypolimnetic phosphorus and iron release from eleven lakes in Maine, USA. **65**: 17.

Berger, E., O. Frör, and R. B. Schäfer. 2019. Salinity impacts on river ecosystem processes: a critical mini-review. Phil. Trans. R. Soc. B **374**: 20180010. doi:10.1098/rstb.2018.0010

Bergmann, M., O. Sobral, J. Pratas, and M. A. S. Graça. 2018. Uranium toxicity to aquatic invertebrates: A laboratory assay. Environmental Pollution **239**: 359–366. doi:10.1016/j.envpol.2018.04.007

Bernhardt, E. S., and G. E. Likens. 2002. Dissolved Organic Carbon Enrichment Alters Nitrogen Dynamics in a Forest Stream. Ecology **83**: 1689–1700. doi:https://doi.org/10.1890/0012-9658(2002)083[1689:DOCEAN]2.0.CO;2

Bernhardt, E. S., and M. A. Palmer. 2011. The environmental costs of mountaintop mining valley fill operations for aquatic ecosystems of the Central Appalachians: Mountaintop mining impacts on aquatic ecosystems. Annals of the New York Academy of Sciences **1223**: 39–57. doi:10.1111/j.1749-6632.2011.05986.x

Best, M. A., A. W. Wither, and S. Coates. 2007. Dissolved oxygen as a physico-chemical supporting element in the Water Framework Directive. Marine Pollution Bulletin **55**: 53–64. doi:10.1016/j.marpolbul.2006.08.037

Bollhöfer, A., J. Brazier, C. Humphrey, B. Ryan, and A. Esparon. 2011. A study of radium bioaccumulation in freshwater mussels, Velesunio angasi, in the Magela Creek catchment, Northern Territory, Australia. Journal of Environmental Radioactivity **102**: 964–974. doi:10.1016/j.jenvrad.2010.04.001

Bormans, M., B. Maršálek, and D. Jančula. 2016. Controlling internal phosphorus loading in lakes by physical methods to reduce cyanobacterial blooms: a review. Aquat Ecol **50**: 407–422. doi:10.1007/s10452-015-9564-x

Boyd, C. E., C. S. Tucker, and B. Somridhivej. 2016. Alkalinity and Hardness: Critical but Elusive Concepts in Aquaculture. Journal of the World Aquaculture Society **47**: 6–41. doi:10.1111/jwas.12241

Cañedo-Argüelles, M., B. J. Kefford, C. Piscart, N. Prat, R. B. Schäfer, and C.-J. Schulz. 2013. Salinisation of rivers: An urgent ecological issue. Environmental Pollution **173**: 157–167. doi:10.1016/j.envpol.2012.10.011

Dai, H. L., K. L. Zhang, X. L. Xu, and H. Y. Yu. 2012. Evaluation on the Effects of Deicing Chemicals on Soil and Water Environment. Procedia Environmental Sciences **13**: 2122–2130. doi:10.1016/j.proenv.2012.01.201

van Dijk, G., L. P. M. Lamers, R. Loeb, P.-J. Westendorp, R. Kuiperij, H. H. van Kleef, M. Klinge, and A. J. P. Smolders. 2019. Salinization lowers nutrient availability in formerly brackish freshwater wetlands; unexpected results from a long-term field experiment. Biogeochemistry **143**: 67–83. doi:10.1007/s10533-019-00549-6

Donnelly, T. H., M. R. Grace, and B. T. Hart. 1997. Algal blooms in the Darling-Barwon River, Australia. Water Air Soil Pollut **99**: 487–496. doi:10.1007/BF02406888

Duan, S., and S. S. Kaushal. 2015. Salinization alters fluxes of bioreactive elements from stream ecosystems across land use. Biogeosciences **12**: 7331–7347. doi:10.5194/bg-12-7331-2015

Duan, S.-W., and S. S. Kaushal. 2013. Warming increases carbon and nutrient fluxes from sediments in streams across land use. Biogeosciences **10**: 1193–1207. doi:10.5194/bg-10-1193-2013

Findlay, S. E. G., and V. R. Kelly. 2011. Emerging indirect and long-term road salt effects on ecosystems. Ann N Y Acad Sci **1223**: 58–68. doi:10.1111/j.1749-6632.2010.05942.x

Galella, J. G., S. S. Kaushal, K. L. Wood, J. E. Reimer, and P. M. Mayer. 2021. Sensors track mobilization of `chemical cocktails’ in streams impacted by road salts in the Chesapeake Bay watershed. Environ. Res. Lett. **16**: 035017. doi:10.1088/1748-9326/abe48f

Geddes, M. 1988. The role of turbidity in the limnology of Lake Alexandrina, River Murray, South Australia; comparisons between clear and turbid phases. Mar. Freshwater Res. **39**: 201. doi:10.1071/MF9880201

Green, S. M., and M. S. Cresser. 2008a. Nitrogen Cycle Disruption through the Application of De-icing Salts on Upland Highways. Water Air Soil Pollut **188**: 139–153. doi:10.1007/s11270-007-9530-x

Green, S. M., and M. S. Cresser. 2008b. Are calcareous soils in uplands less prone to damage from road salting than acidic soils? Chemistry and Ecology **24**: 1–13. doi:10.1080/02757540701814614

Green, S. M., R. Machin, and M. S. Cresser. 2008a. Effect of long-term changes in soil chemistry induced by road salt applications on N-transformations in roadside soils. Environmental Pollution **152**: 20–31. doi:10.1016/j.envpol.2007.06.005

Green, S. M., R. Machin, and M. S. Cresser. 2008b. Long-term road salting effects on dispersion of organic matter from roadside soils into drainage water. Chemistry and Ecology **24**: 221–231. doi:10.1080/02757540802032181

Grosell, M., J. Blanchard, K. V. Brix, and R. Gerdes. 2007. Physiology is pivotal for interactions between salinity and acute copper toxicity to fish and invertebrates. Aquatic Toxicology **84**: 162–172. doi:10.1016/j.aquatox.2007.03.026

Hansson, D., and E. Gustafsson. 2011. Salinity and hypoxia in the Baltic Sea since A.D. 1500. Journal of Geophysical Research: Oceans **116**. doi:10.1029/2010JC006676

Haq, S., S. S. Kaushal, and S. Duan. 2018. Episodic salinization and freshwater salinization syndrome mobilize base cations, carbon, and nutrients to streams across urban regions. Biogeochemistry **141**: 463–486. doi:10.1007/s10533-018-0514-2

Iglesias, M. C.-A. 2020. A review of recent advances and future challenges in freshwater salinization. Limnetica **39**: 185–211.

Jeppesen, E., S. Brucet, L. Naselli-Flores, and others. 2015. Ecological impacts of global warming and water abstraction on lakes and reservoirs due to changes in water level and related changes in salinity. Hydrobiologia **750**: 201–227. doi:10.1007/s10750-014-2169-x

Judd, J. H. 1970. Lake stratification caused by runoff from street deicing. Water Research **4**: 12.

Kaushal, S. S., S. Duan, T. R. Doody, and others. 2017. Human-accelerated weathering increases salinization, major ions, and alkalinization in fresh water across land use. Applied Geochemistry **83**: 121–135. doi:10.1016/j.apgeochem.2017.02.006

Kaushal, S. S., A. J. Gold, S. Bernal, and others. 2018a. Watershed ‘chemical cocktails’: forming novel elemental combinations in Anthropocene fresh waters. Biogeochemistry **141**: 281–305. doi:10.1007/s10533-018-0502-6

Kaushal, S. S., G. E. Likens, M. L. Pace, and others. 2019. Novel ‘chemical cocktails’ in inland waters are a consequence of the freshwater salinization syndrome. Phil. Trans. R. Soc. B **374**: 20180017. doi:10.1098/rstb.2018.0017

Kaushal, S. S., G. E. Likens, M. L. Pace, and others. 2021. Freshwater salinization syndrome: from emerging global problem to managing risks. Biogeochemistry. doi:10.1007/s10533-021-00784-w

Kaushal, S. S., G. E. Likens, M. L. Pace, R. M. Utz, S. Haq, J. Gorman, and M. Grese. 2018b. Freshwater salinization syndrome on a continental scale. Proc Natl Acad Sci USA **115**: E574–E583. doi:10.1073/pnas.1711234115

Kaushal, S. S., P. M. Mayer, P. G. Vidon, and others. 2014. Land Use and Climate Variability Amplify Carbon, Nutrient, and Contaminant Pulses: A Review with Management Implications. JAWRA Journal of the American Water Resources Association **50**: 585–614. doi:https://doi.org/10.1111/jawr.12204

Lazur, A., T. VanDerwerker, and K. Koepenick. 2020. Review of Implications of Road Salt Use on Groundwater Quality—Corrosivity and Mobilization of Heavy Metals and Radionuclides. Water Air Soil Pollut **231**: 474. doi:10.1007/s11270-020-04843-0

Li, X., Z. Rengel, E. Mapfumo, and Bhupinderpal-Singh. 2007. Increase in pH stimulates mineralization of ‘native’ organic carbon and nitrogen in naturally salt-affected sandy soils. Plant Soil **290**: 269–282. doi:10.1007/s11104-006-9158-4

Liang, Y., R. Y. H. Cheung, and M. H. Wong. 1999. Reclamation of wastewater for polyculture of freshwater fish: bioaccumulation of trace metals in fish. Water Research **33**: 2690–2700. doi:10.1016/S0043-1354(98)00473-4

Lind, L., M. S. Schuler, W. D. Hintz, A. B. Stoler, D. K. Jones, B. M. Mattes, and R. A. Relyea. 2018. Salty fertile lakes: how salinization and eutrophication alter the structure of freshwater communities. Ecosphere **9**: e02383. doi:https://doi.org/10.1002/ecs2.2383

Marton, J. M., E. R. Herbert, and C. B. Craft. 2012. Effects of Salinity on Denitrification and Greenhouse Gas Production from Laboratory-incubated Tidal Forest Soils. Wetlands **32**: 347–357. doi:10.1007/s13157-012-0270-3

Mavi, M. S., J. Sanderman, D. J. Chittleborough, J. W. Cox, and P. Marschner. 2012. Sorption of dissolved organic matter in salt-affected soils: Effect of salinity, sodicity and texture. Science of The Total Environment **435–436**: 337–344. doi:10.1016/j.scitotenv.2012.07.009

Mclaughlin, M., K. Tiller, R. Naidu, and D. Stevens. 1996. Review: the behaviour and environmental impact of contaminants in fertilizers. Soil Res. **34**: 1. doi:10.1071/SR9960001

McNaboe, L. A., G. A. Robbins, and M. E. Dietz. 2017. Mobilization of Radium and Radon by Deicing Salt Contamination of Groundwater. Water Air Soil Pollut **228**: 94. doi:10.1007/s11270-016-3227-y

Mirlean, N., S. T. Larned, V. Nikora, and V. Tavares Kütter. 2005. Mercury in lakes and lake fishes on aconservation-industry gradient in Brazil. Chemosphere **60**: 226–236. doi:10.1016/j.chemosphere.2004.12.047

Nazarbeygi, E., H. Yazdi, R. Naseri, and R. Soleimani. 2011. The Effects of Different Levels of Salinity on Proline and A-, B-Chlorophylls in Canola. **10(1)**.

Nielsen, D. L., M. A. Brock, G. N. Rees, and D. S. Baldwin. 2003. Effects of increasing salinity on freshwater ecosystems in Australia. Aust. J. Bot. **51**: 655–665. doi:10.1071/bt02115

Norrström, A. C., and E. Bergstedt. 2001. The Impact of Road De-Icing Salts (NaCl) on Colloid Dispersion and Base Cation Pools in Roadside Soils. Water Air Soil Pollut **127**: 19.

de Oliveira-Filho, E. C., R. M. Lopes, and F. J. R. Paumgartten. 2004. Comparative study on the susceptibility of freshwater species to copper-based pesticides. Chemosphere **56**: 369–374. doi:10.1016/j.chemosphere.2004.04.026

Ramakrishna, D. M., and T. Viraraghavan. 2005. Environmental Impact of Chemical Deicers – A Review. Water Air Soil Pollut **166**: 49–63. doi:10.1007/s11270-005-8265-9

Riedel, T., and C. Kübeck. 2018. Uranium in groundwater – A synopsis based on a large hydrogeochemical data set. Water Research **129**: 29–38. doi:10.1016/j.watres.2017.11.001

Sahrawat, K. L. 2008. Factors Affecting Nitrification in Soils. Communications in Soil Science and Plant Analysis **39**: 1436–1446. doi:10.1080/00103620802004235

Sandor, Z., I. Csengeri, M. B. Oncsik, M. N. Alexis, and E. Zubcova. 2001. Trace metal levels in freshwater fish, sediment and water. Environ Sci & Pollut Res **8**: 265–268. doi:10.1007/BF02987404

Sauer, F. G., M. Bundschuh, J. P. Zubrod, R. B. Schäfer, K. Thompson, and B. J. Kefford. 2016. Effects of salinity on leaf breakdown: Dryland salinity versus salinity from a coalmine. Aquatic Toxicology **177**: 425–432. doi:10.1016/j.aquatox.2016.06.014

Sherwood, J. E., F. Stagnitti, M. J. Kokkinn, and W. D. Williams. 1991. Dissolved oxygen concentrations in hypersaline waters. Limnology and Oceanography **36**: 235–250. doi:10.4319/lo.1991.36.2.0235

Šimek, M., and J. E. Cooper. 2002. The influence of soil pH on denitrification: progress towards the understanding of this interaction over the last 50 years. European Journal of Soil Science **53**: 345–354. doi:https://doi.org/10.1046/j.1365-2389.2002.00461.x

Smolders, A. J. P., L. P. M. Lamers, E. C. H. E. T. Lucassen, G. V. D. Velde, and J. G. M. Roelofs. 2006. Internal eutrophication: How it works and what to do about it—a review. Chemistry and Ecology **22**: 93–111. doi:10.1080/02757540600579730

Spiteri, C., P. V. Cappellen, and P. Regnier. 2008. Surface complexation effects on phosphate adsorption to ferric iron oxyhydroxides along pH and salinity gradients in estuaries and coastal aquifers. Geochimica et Cosmochimica Acta **72**: 3431–3445. doi:10.1016/j.gca.2008.05.003

Steele, M. K., and J. A. Aitkenhead-Peterson. 2011. Long-term sodium and chloride surface water exports from the Dallas/Fort Worth region. Science of The Total Environment **409**: 3021–3032. doi:10.1016/j.scitotenv.2011.04.015

Tripler, C. E., S. S. Kaushal, G. E. Likens, and M. T. Walter. 2006. Patterns in potassium dynamics in forest ecosystems. Ecology Letters **9**: 451–466. doi:https://doi.org/10.1111/j.1461-0248.2006.00891.x

White, D. A., A. Pagarette, P. Rooks, and S. T. Ali. 2013. The effect of sodium bicarbonate supplementation on growth and biochemical composition of marine microalgae cultures. J Appl Phycol **25**: 153–165. doi:10.1007/s10811-012-9849-6

Yue, L., W. Kong, C. Li, G. Zhu, L. Zhu, T. P. Makhalanyane, and D. A. Cowan. 2021. Dissolved inorganic carbon determines the abundance of microbial primary producers and primary production in Tibetan Plateau lakes. FEMS Microbiology Ecology **97**. doi:10.1093/femsec/fiaa242

**Figure 5.**

Data illustrated in Fig. 5 (Bottom Panel) were derived from automated sensors deployed by the United States Geological Survey (USGS). The data for each site can be downloaded from the USGS National Water Information System. Sites shown include the [Northeast Branch Anacostia River (USGS gage number 01649500)](https://waterdata.usgs.gov/dc/nwis/uv/?site_no=01651000&PARAmeter_cd=00065,00060,62620), [Capacon River (01611500)](https://waterdata.usgs.gov/usa/nwis/uv?01611500), [Difficult Run (01646000)](https://waterdata.usgs.gov/usa/nwis/uv?01646000), and the [Potomac River (01646500)](https://waterdata.usgs.gov/usa/nwis/uv?01646500).
